# The application value of Rs-fMRI-based machine learning models for differentiating mild cognitive impairment from Alzheimer's disease: a systematic review and meta-analysis

**DOI:** 10.1007/s10072-024-07731-1

**Published:** 2024-09-03

**Authors:** Chentong Wang, Li Zhou, Feng Zhou, Tingting Fu

**Affiliations:** 1 Rheumatology Immunology Department, Ningbo Medical Center Lihuili Hospital, Ningbo, Zhejiang 315000 China; 2 Ningbo Medical Center Lihuili Hospital, 1111 Jiangnan Road, Yinzhou District, Ningbo, Zhejiang China

**Keywords:** Machine Learning, Deep Learning, Imaging Histology, Resting-State Functional MRI, Mild Cognitive Impairment, Alzheimer's Disease

## Abstract

**Background:**

Various machine learning (ML) models based on resting-state functional MRI (Rs-fMRI) have been developed to facilitate differential diagnosis of mild cognitive impairment (MCI) and Alzheimer's disease (AD). However, the diagnostic accuracy of such models remains understudied. Therefore, we conducted this systematic review and meta-analysis to explore the diagnostic accuracy of Rs-fMRI-based radiomics in differentiating MCI from AD.

**Methods:**

PubMed, Embase, Cochrane, and Web of Science were searched from inception up to February 8, 2024, to identify relevant studies. Meta-analysis was conducted using a bivariate mixed-effects model, and sub-group analyses were carried out by the types of ML tasks (binary classification and multi-class classification tasks).

**Findings:**

In total, 23 studies, comprising 5,554 participants were enrolled in the study. In the binary classification tasks (twenty studies), the diagnostic accuracy of the ML model for AD was 0.99 (95%CI: 0.34 ~ 1.00), with a sensitivity of 0.94 (95%CI: 0.89 ~ 0.97) and a specificity of 0.98 (95%CI: 0.95 ~ 1.00). In the multi-class classification tasks (six studies), the diagnostic accuracy of the ML model was 0.98 (95%CI: 0.98 ~ 0.99) for NC, 0.96 (95%CI: 0.96 ~ 0.96) for early mild cognitive impairment (EMCI), 0.97 (95%CI: 0.96 ~ 0.97) for late mild cognitive impairment (LMCI), and 0.95 (95%CI: 0.95 ~ 0.95) for AD.

**Conclusions:**

The Rs-fMRI-based ML model can be adapted to multi-class classification tasks. Therefore, multi-center studies with large samples are needed to develop intelligent application tools to promote the development of intelligent ML models for disease diagnosis.

**Supplementary Information:**

The online version contains supplementary material available at 10.1007/s10072-024-07731-1.

## Introduction

**Fig. 1:**
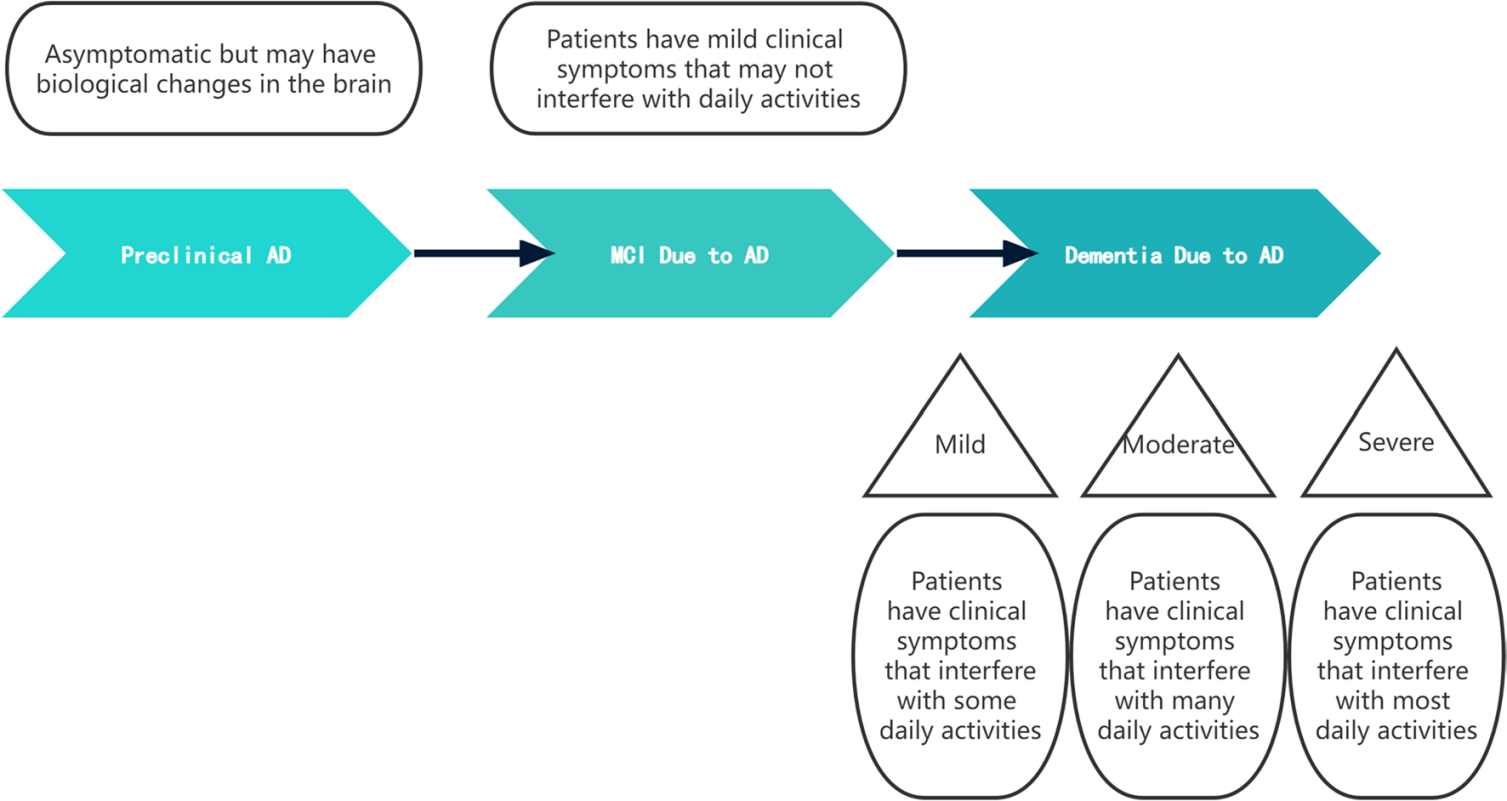
Disease processes in AD

Alzheimer's disease (AD) is a progressive neurodegenerative disorder primarily affecting the brain. The disease manifests with neuroinflammatory plaques, neurogenic fiber tangles, neuronal loss and amyloid angiopathy. It is the most common cause of dementia, accounting for an estimated 60 to 80% of patients [1]. Clinically, its early symptoms include difficulty remembering recent conversations, names or events, apathy, and depression. Some patients may exhibit communication problems, confusion, poor judgment, and behavioral changes. In the advanced stages of AD, patients may develop complications such as walking, talking and swallowing [1]. The development of AD has three phases: preclinical AD, MCI due to AD, and dementia due to AD, also known as AD dementia (Fig. 1). The pre-symptomatic dementia stage of AD is known as AD-induced mild cognitive impairment (MCI). The AD stage occurs when the patient experiences a gradual progressive decline in cognitive function due to advancement in AD pathology in the brain. Significant cognitive impairment may interfere with daily functioning leading to AD dementia [2]. This pathological process is also known as the AD continuum and typically starts with subtle changes in the brain, which are not immediately noticeable [3]. MCI can be further categorized into early mild cognitive impairment (EMCI) and late mild cognitive impairment (LMCI) [4], with EMCI presenting with milder episodic memory impairment compared with LMCI. Some studies have shown that the risk of LMCI progression to AD is much higher than that of EMCI [5, 6]. The latest Global Burden of Disease study states that AD is the fifth leading cause of death worldwide [7]. In the United States, approximately 6.5 million people over the age of 65 years are affected by AD [8]. Considering the lack of interventions to prevent, slow down, or cure AD, this number is expected to increase to 13.8 million by 2060 [9]. Consequently, to prevent its detrimental effect on the society, researchers should aim to develop a strategy or intervention to control MCI, thereby reverse or delay AD. In population-based studies, an early systematic review and meta-analysis reported a 26% reversal rate [10]. This underscores the need to explore the transition from MCI to AD.

Several tools, such as computed tomography (CT), positron emission tomography (PET), structural magnetic resonance imaging (sMRI), resting-state functional magnetic resonance imaging (Rs-fMRI), and cerebrospinal fluid analysis of β-amyloid (Aβ) or tau protein (total tau [T-tau] and phosphorylated tau [P-tau]) concentrations have been suggested as potential diagnostic methods for AD. These techniques allow clinicians to comprehensively assess the brain structure and function in patients with AD at the molecular level, cellular level, metabolic level, and from a microcirculatory perspective [11]. However, these tools are currently in the exploratory stage and have various limitations in clinical application. For instance, cerebrospinal fluid (CSF) biomarker testing is clinically unacceptable to patients due to its invasive nature. CT does not accurately examine the subtle changes in brain structure and functions, and is therefore not ideal for early AD [12]. In comparison, PET can detect pathological changes in the nervous system at an early stage and is suitable for early diagnosis of AD. However, the specificity of PET in differentiating AD and MCI is poor [13], and it is unable to accurately identify AD at the subclinical stage. Although sMRI can detect structural changes in the brain, such alterations are not always fully consistent with pathologic observations in AD patients. These findings reveal various limitations in evaluating disease severity and prognosis [14]. Rs-fMRI is used to assess local brain activity and resting-state networks. It circumvents the performance problems inherent in task-state functional magnetic resonance studies [15]. The core principle of resting-state fMRI (rs-fMRI) involves quantifying fluctuations in blood-oxygen-level-dependent (BOLD) signals, which are linked to regional changes in blood flow and oxygen metabolism. These fluctuations provide indirect measures of spontaneous neuronal activity within the brain [16]. Early diagnosis of AD is critical as it allows effective treatment and improves the prognosis of AD patients. Rs-fMRI can identify cognitive changes by detecting and evaluating changes in brain structure and function in a non-invasive manner, making it suitable for early AD and differentiating MCI from AD [17, 18]. Neuroimaging techniques described above require specialized knowledge and experience in interpreting the results. This limits their widespread use in clinical practice, especially in economically underdeveloped regions. Artificial intelligence (AI) tools can analyze the data in brain images and make a diagnosis automatically through techniques such as machine learning (ML) and deep learning (DL) [19]. The AI system outperforms general radiologists and imaging interns in conducting the differential diagnosis of brain diseases, often providing accuracy that matches that of academic neuroradiologists [20]. Therefore, to generate ideas to improve the diagnosis and prevention of AD, we conducted this study to evaluate the significance of rs-fMRI combined with AI techniques [21].

This study aimed to systematically review evidence regarding the clinical value of Rs-fMRI-based ML models in differentiating MCI from AD. Specifically, we explored the accuracy, sensitivity, and specificity of these models, as well as their utility in clinical practice. The findings of this analysis will improve early diagnosis of MCI and AD through the use of Rs-fMRI-based AI learning models. They will also accelerate the establishment of a more personalized treatment plan for AD patients. The results may also provide valuable information about the pathogenesis of MCI and AD. Finally, the study will provide ideas for developing advanced Rs-fMRI-based AI learning models for the diagnosis and treatment of other neurodegenerative diseases.

## Methods

### Study registration

This systematic review was conducted in line with the Preferred Reporting Items for a Systematic Review and Meta-analysis of Diagnostic Test Accuracy Studies (The PRISMA-DTA Statement) and has been prospectively registered with PROSPERO (CRD42023421722).

### Eligibility criteria

#### Inclusion criteria

The patients included in this systematic review had complete Rs-fMRI images and were diagnosed with MCI and AD. The types of studies included were cohort studies, case–control studies, and cross-sectional studies. We selected studies that were highly relevant to the topic of this meta-analysis in terms of research questions, interventions, and outcome indicators. To ensure that the results were comprehensive and generalizable, the enrolled studies were from diverse research backgrounds, cultures, and regions. Studies were considered eligible if they provided sufficient data for this meta-analysis, included necessary demographic information, interventions, and outcome measures.

#### Exclusion criteria

The following studies were excluded:

- (1) Meta-analyses, reviews, guidelines, expert opinions, etc.
- (2) Studies in which only risk factor analysis was performed and no complete ML model was constructed;
- (3) Studies without outcomes such as recall, Roc, F1 score, c-statistic, c-index, sensitivity, specificity, accuracy, precision, confusion matrix, diagnostic tetrad, and calibration curve in the assessment of accuracy of ML models.
- (4) Validation studies of mature scales only.
- (5) Studies using a single factor to determine prediction accuracy.

### Data sources and search strategy

A systematic search was conducted on PubMed, Cochrane, Embase, and Web of Science to identify relevant studies from the inception of these databases up to February 25, 2023. To ensure the inclusion of all relevant studies, we conducted an updated database search on February 8, 2024. The search strategy combined subject terms and free-text keywords with no geographic restrictions. The detailed search strategy is provided in Table S1.

### Study selection and data extraction

The identified literature were imported into Zotero followed by exclusion of duplicates using automatic software tagging and manual tagging. Next, the studies were screened by reading the titles and abstracts. Subsequently, we downloaded the full texts of the remaining studies, and conducted further screening by reading the full texts of the studies. Before data extraction, we developed a standardized data extraction spreadsheet, containing study title, country, year of publication, first author, study type, patient source, diagnostic criteria, number of cases, validation method, and model type.

The study selection and data extraction were performed independently by two researchers. The screening and extraction results were cross-checked and in cases of disputes, a third researcher was consulted to reach a consensus.

### Risk of bias assessment

The methodological quality of the included studies was evaluated using the Radiomics Quality Score (RQS) and the results were cross-checked by two independent investigators. Any disputes were addressed by consulting a third researcher. In addition, we employed the RQS, a radiomics-specific quality assessment tool, to evaluate the methodological quality of the original study design across 16 of the 36 study components.

### Outcomes

The included original studies were categorized into binary classification and multi-class classification tasks based on the type of ML task. For binary classification tasks, the outcome metrics were sensitivity, positive likelihood ratio (PLR), negative likelihood ratio (NLR), specificity, diagnostic odds ratio (DOR), and summary ROC curve (SROC). For the multi-class classification tasks, the outcome metrics were diagnostic accuracy for each category.

### Synthesis methods

Statistical analysis was conducted using Stata 15.0. The c-statistic was used as the systematic evaluation index to evaluate the accuracy evaluation of the model. C-statistic and its 95% confidence interval were calculated in the meta-analysis. A random effects model was used to complete the meta-analysis of the c-statistic. Moreover, we utilized a bivariate mixed-effects model to combine the sensitivity and specificity of the model predictions to and prevent the effect of severe data imbalance on the prediction accuracy. A *p* value < 0.05 was considered statistically significant.

## Results

### Study selection

**Fig. 2:**
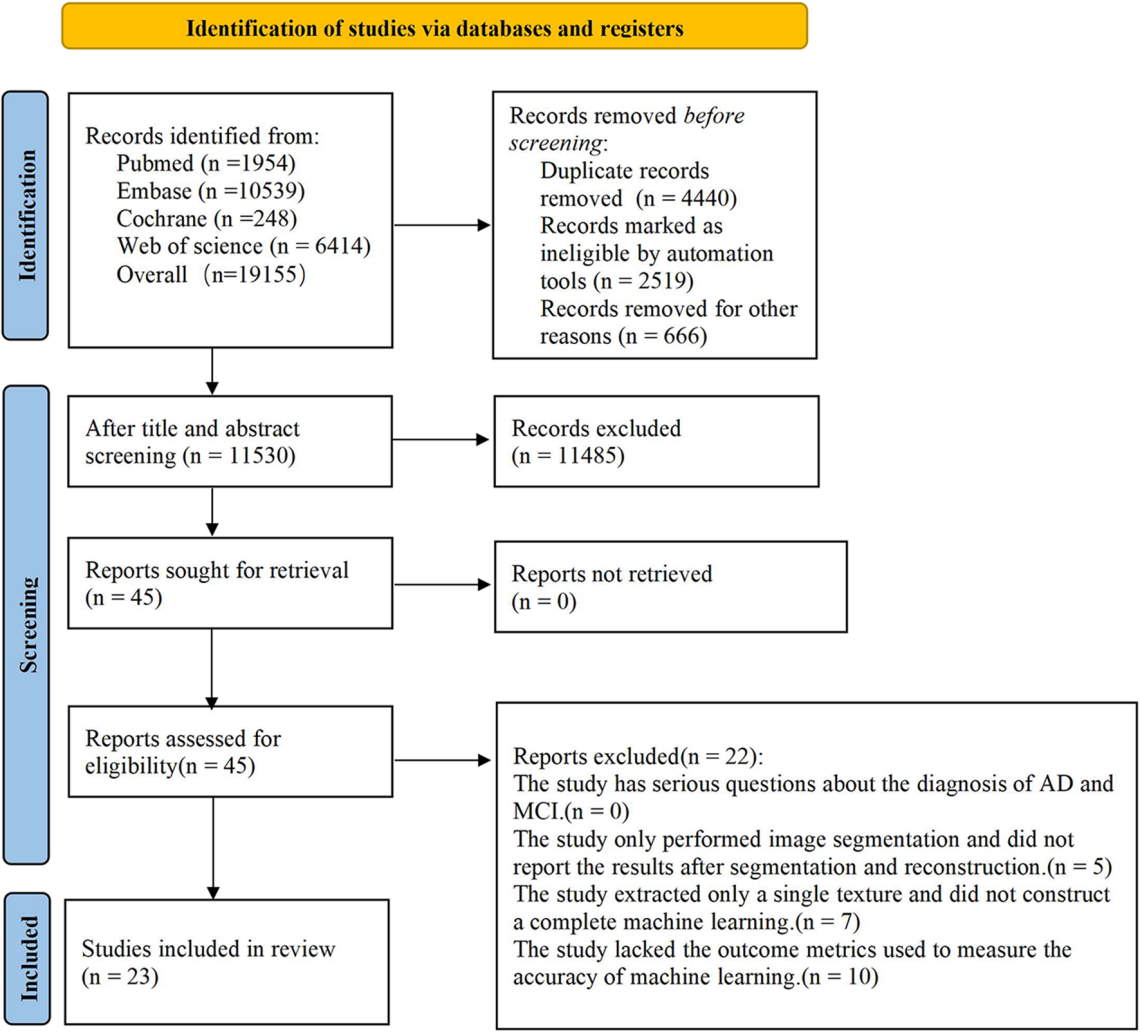
Literature screening process

The search retrieved 15,870 articles up to February 25, 2023. Subsequently, we updated the search for all relevant studies in the databases up to February 8, 2024 to comprehensively include relevant literature and enhance the reliability of the meta-analysis results. We initially retrieved 19,155 records, from which we identified 4,440 duplicates. After removing duplicates, we screened titles and abstracts to select 45 relevant studies for full-text review. Ultimately, 23 studies met the inclusion criteria for this study [22–44]. (Fig. 2).

### Study characteristics

A total of 23 studies were included in this study, comprising 5,554 participants. The included studies were case–control investigations. Two of the included studies were conducted in Canada, four in China, one in Egypt, one in Germany, one in India, three in Iran, four in Korea, one in Lithuania, one in Pakistan, one in Singapore, and four in the United States. The included literature comprised 12 articles on machine learning and 13 on deep learning. Regarding classification tasks, 20 articles focused on binary classification, while six addressed multi-class classification. Among the included studies, none performed hyperparameter optimization for the reported DL and ML models. (Table 1).

**Table 1:** Clinical characteristics of the included studies

NO	First author	Year of publication	Country	Patient source	Diagnostic criteria for mild cognitive impairment	Diagnostic criteria for AD	Total number of Alzheimer's disease cases	Total number of MCI	Total number of cases	Generation of validation set	Number of Alzheimer's disease cases in training set	Number of MCI cases in training set	Number of cases in training set	Number of cases in validation set	Number of cases in test set	Model type
1	Yosra Kazemi	2018	Canada	ADNI	MMSE	MMSE	29	85	197	fivefold cross validation	29	85	197			AlexNet
2	Yu Wang	2019	China	ADNI database			34	35	104	tenfold cross validation	34	35	104			SVM, KNN
3	Bocheng Wang	2022	China	ADNI2 dataset	CDR and MMSE	CDR and MMSE	30	87	160	random sampling			114	16	30	
4	SAMAN SARRAF	2016	Canada	ADNI	MMSE	MMSE	52	131	275	fivefold cross validation	52	131	275			MCADNNet (an optimized convolutional neural network (CNN) topology)
5	Modupe Odusami	2021	Lithuania	ADNI			25	63	138	random sampling			51,443 (number of images, 70% for training and 30% for validation in each group)	27,310		ResNet18(Residual Network)
6	ZHE WANG	2018	USA	Singe center	Petersen criteria	NINCDS-ADRDA criteria	10	11	33	Leave-One-Out cross-validation (LOOCV)						AdaBoost
7	Harshit Parmar	2020	United States	ADNI			30	60	120	five-fold cross validation						3D CNN
8	Konrad F. Waschkies	2022	Germany	Multi-center	The performance on the "recall word list" subtest in CERAD was worse than average (> 1.5 SD) CERAD	MMSE	74	132	733	tenfold cross validation						multi-class support vector machine
9	PR. Buvaneswari	2021	India	ADNI	MMSE score between 25–32; Wechsler Memory Scale Logical Memory II score 23–29; CDR 0.5; no significant impairment in other cognitive areas; no dementia	MMSE:20–30; CDR: 0.5–1.0	68	69	210	Unclear						kernel-SVR method, kSVR
10	Nazanin Beheshti	2022	USA	ADNI and OASIS			250	97	648	random sampling						CNN and Transformer
11	Sukrit Gupta	2019	Singapore	ADNI	Subjects classified as MCI have memory impairment, objective memory loss as measured by the education-adjusted score on the Wechsler Logistic Memory Scale Memory II, with no dementia and severe impairment in other cognitive domains	The criteria for AD by National Institute of Neurological and Communicative Disorders and Stroke and the Alzheimer’s Disease and Related Disorders Association	29	90	168	fivefold cross validation						5-layer feed-forward deep neural network (DNN)
12	Bohyun Wang	2022	Korea	ADNI			34	89	168	a hold-out verification			23		99	ZNN
13	Doaa Mousa	2022	Egypt	ADNI			114	231	512	tenfold cross validation						SVM
14	Tingting Zhang	2021	China	ADNI	The diagnostic criteria for MCI are as follows: (1) MMSE score between 24 and 30. (2) CDR is 0.5. (3) Memory complaints, objective memory loss measured by scores on the Educationally Adjusted Wechsler Memory Scale Logical Memory II. (4) There is no obvious impairment in other cognitive areas and the patient can remember activities of daily living (no dementia)		19	85	104	cross validation						SVM classifier
15	Mohammadmahdi Rahimiasl	2021	Iran	ADNI	The studies (Xue et al., 2019; Zhang et al., 2019) provide more information on the data collection protocol of ADNI and the diagnostic criteria for AD, MCI and HC		26	63	125	fivefold cross validation						L2 regularization logistic regression and linear SVM classifier
16	Farheen Ramzan	2020	Pakistan	ADNI	Cognitive testing (i.e.,MMSE) and CDR	Cognitive testing (i.e.,MMSE) and CDR	25	63	138	random sampling			70% ( 595,056 images)	20%(170,016 images)	10%(85,005 images)	ResNet18(Residual Network)
17	Yubraj Gupta	2020	South Korea	ADNI	MCIs group: MMSE score 25–30 points, FAQ: 0–16 points, and GDS: 0–13 points MCIc group: MMSE: 19–30 points, FAQ: 0–18 points, GDS: 0–10 points	AD group: CDR score: 1 point, the MMSE score: 14–24 points, the FAQ score: 3–28 points; GDS score: 0–7 points	33	61	129	LOOCV			70%(compared to the test set)		30%	MKL algorithm classifier(Multiple Kernel Learning)
18	Seong-Jin Son	2017	Korea	ADNI	CDR score: 0.5, MMSE score: 24–30	CDR score: 0.5, MMSE score: 24–30	30	40	105	LOOCV						random forest classifier
19	Ali Khazaee	2016	Iran	ADNI	MMSE score: 24–30, memory complaints, objective memory loss as measured by the educationally adjusted score of the Wechsler Memory Scale Logical Memory II, CDR: 0.5, no significant impairment in other cognitive areas, basically retained activities of daily living, and no dementia "	MMSE score: 14–26 points, CDR: 0.5 or 1.0, and those mmet the diagnostic criteria for AD in the National Institute of Neurological and Communicative Disorders and Stroke and Alzheimer's Disease and Related Disorders Society (NINCDS/ ADRDA)	34	89	168	tenfold cross validation						naïve Bayesian classifier
20	Ali Khazaee	2015	Iran	ADNI	Patients with AD have a MMSE score of 14–26 and a CDR of 0.5 or 1.0, and meet the possible diagnostic standards for AD in the National Institute of Neurological and Communicative Disorders and Stroke and the Alzheimer's and Related Disorders Association (NINCDS/ADRDA)	Patients with MCI have a MMSE scores of 24–30, memory complaints, objective memory loss (measured by educationally adjusted scores on the Wechsler Memory Scale Logical Memory II), a CDR of 0.5, have no severe impairment in other cognitive areas, largely preserve activities of daily living. and have no dementia	34	89	168	Holdout cross validation	23	23	23		95	SVM
21	Zhuqing Long	2023	China	Singe center	CDR, MMSE and AVLT	CDR, MMSE and AVLT	44	66	168	tenfold cross validation						SVM
22	Saman Sarraf	2023	USA	ADNI	MMSE	MMSE	54	131	284	random sampling			226	27	31	CNN
23	Ju-Hyeon Noh	2023	Korea	ADNI	MMSE	MMSE	118	397	699	fivefold cross validation						3D-CNN

*CDR* Clinical dementia rating scale, *MMS* Mini-mental state exam, *AVLT* Auditory verbal learning test

### Risk of bias assessment

**Fig. 3:**
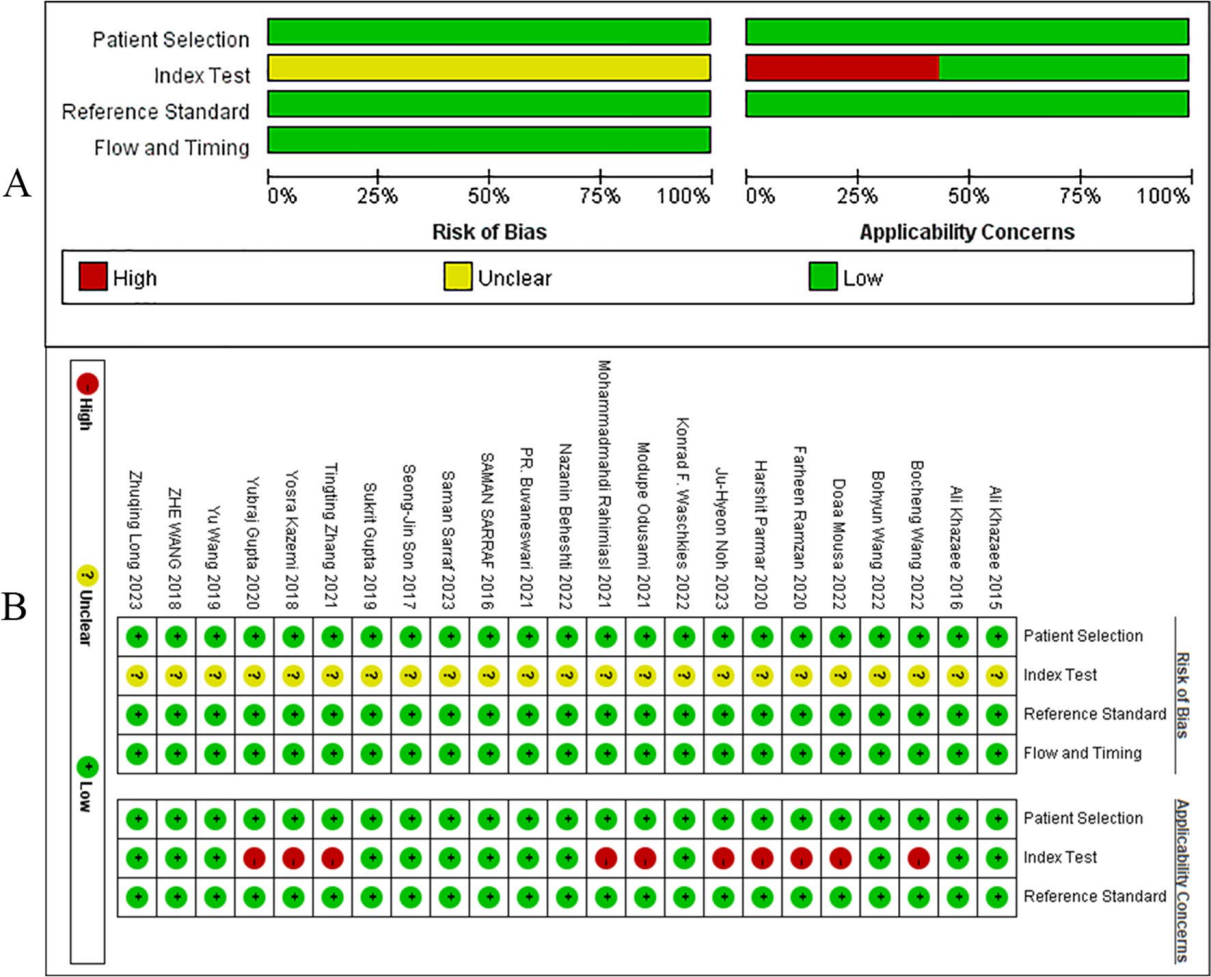
**A** Risk of bias assessment-Methodological quality graph; **B** Risk of bias assessment-Methodological quality summary

The risk of bias in the included studies was assessed using the Quality Assessment of Diagnostic Accuracy Studies-2 (QUADAS-2) scale [45]. All the included studies used the method of Rs-fMRI alone to classify the diseases, and there were no other measures to interfere with the classification accuracy of the model. Most included studies employed a case–control design, which can introduce population bias. Additionally, the potential for publication bias exists, as studies with positive findings may be more likely to be published than those with negative results. Furthermore, two studies from the same research group were included, which could impact the overall results. Regarding disease diagnosis, all studies used clinical scales as the basis for sample inclusion. Diagnoses such as CSF or imaging biomarkers are not included. Therefore, this clinical diagnosis-based MCI/AD cohort may have biased the results. In addition, there may be bias arising from a literature database. Most of the case data in the literature were retrieved from the ADNI database. Currently, there is no single database that comprehensively host all published medical literature, and the criteria for inclusion vary from country to country, potentially leading to biased results in systematic evaluations. Evaluating publication bias is crucial for accurately understanding research findings, informing clinical practice, enhancing study design, improving research quality, and promoting research transparency.(Fig. 3 and Table S2).

## Meta analysis

### Binary classification tasks

#### Synthesized results

**Fig. 4:**
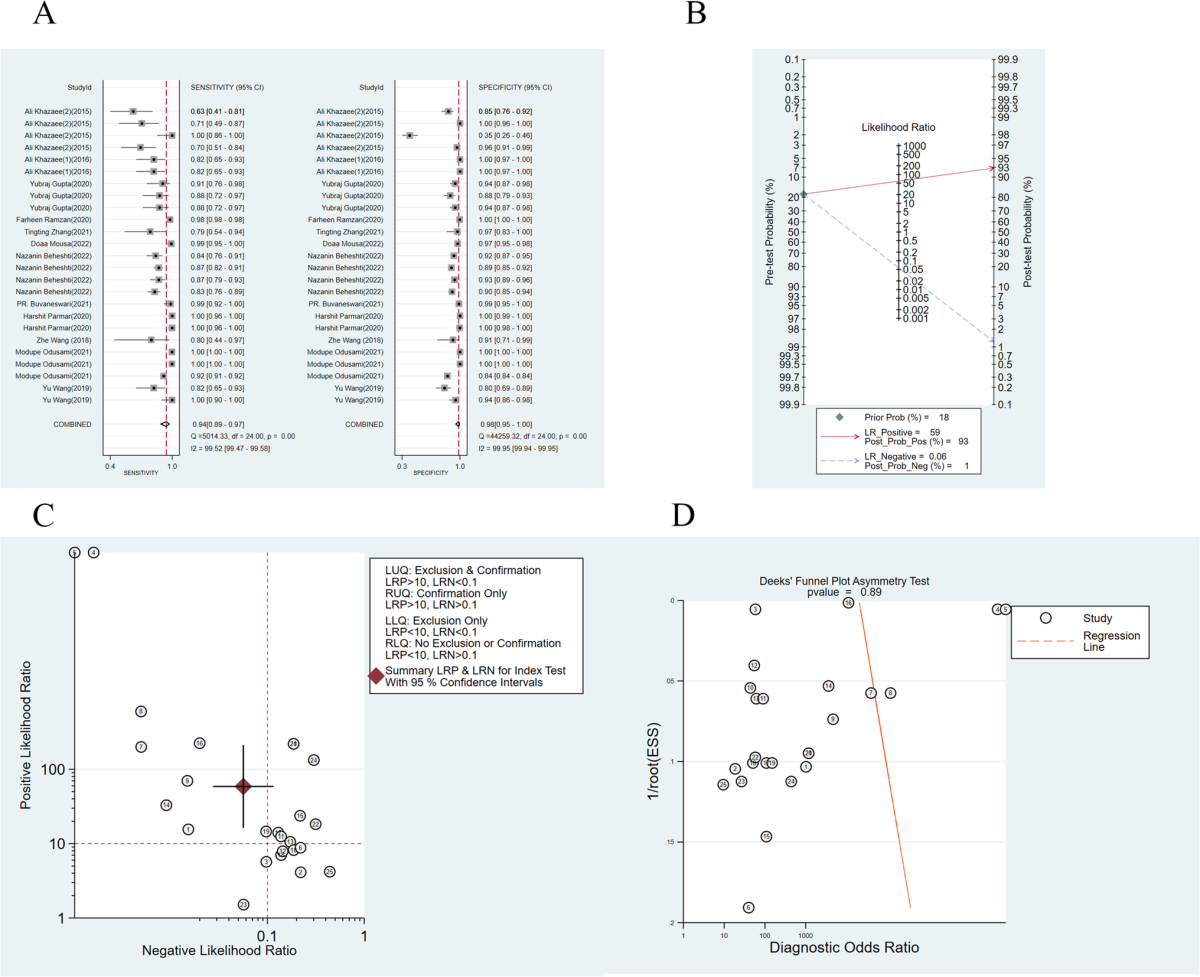
**A** Forest plot of meta-analysis of total diagnostic accuracy of AD in binary classification tasks; **B** Column-line plot of meta-analysis of total diagnostic accuracy of AD in binary classification tasks; **C** Quadrant plot of meta-analysis of total diagnostic accuracy of AD in binary classification tasks; **D** Deeks’ funnel plot of meta-analysis of total diagnostic accuracy of AD in binary classification tasks

Among the included studies, 20 reported binary classification tasks involving 25 models. The binary classification tasks had an average sensitivity of 0.94 (95%CI: 0.89 ~ 0.97), a specificity of 0.98 (95%CI: 0.95 ~ 1.00), a PLR of 58.8 (95%CI: 16.6 ~ 208.6), an NLR of 0.06 (95%CI: 0.03 ~ 0.11), a DOR of 1041 (95%CI: 169 ~ 6425), and an AUROC of 0.99 (95%CI: 0.34 ~ 1.00) (Fig. 4).

The percentage of AD in our included population was about 18%. Its clinical applicability was evaluated using a column-line plot. We utilized the 18% prevalence as a priori probability that in the case of a positive ML result, there is a 93% chance that the presence of AD is real, and if the ML result is negative, there is only a 1% chance that the presence of AD is real.

Of the constructed models, nine exhibited strong diagnostic performance for both AD diagnosis and exclusion, with positive and negative likelihood ratios of 10 and 0.1, respectively. Another seven models effectively diagnosed AD but had limitations in excluding it, while two models excelled at exclusion but struggled with diagnosis. The remaining six models demonstrated suboptimal performance in both diagnostic tasks.

Analysis of the Deeks’ funnel plot revealed no publication bias (*p* = 0.89).

### Subgroup analysis

Subgroup analysis was conducted by the type of validation and type of modeling in the included studies.

### Model type

#### Deep learning

The DL models showed a sensitivity of 0.96 (95%CI: 0.91 ~ 0.99), a specificity of 0.99 (95%CI: 0.94 ~ 1.00), a PLR of 100.9 (95%CI: 14.6 ~ 697.0), an NLR of 0.04 (95%CI: 0.01 ~ 0.10), a DOR of 2853 (95%CI: 159 ~ 51220), and an AUROC of 0.99 (95%CI: 0.56 ~ 1.00). (Figure S1 and S2).

#### Machine learning

ML models had a sensitivity of 0.91 (95%CI: 0.78 ~ 0.97), a specificity of 0.97 (95%CI: 0.89 ~ 0.99), a PLR of 32.6 (95%CI: 8.0 ~ 132.6), an NLR of 0.09 (95%CI: 0.03 ~ 0.23), a DOR of 361 (95%CI: 74 ~ 1756), and an AUROC of 0.98 (95%CI: 0.33 ~ 1.00). (Figure S3 and S4).

Although ML demonstrated slightly lower overall predictive performance compared to DL, it still yields promising results. The SVMs represent one of the more effective ML methods. The SVM models had a sensitivity of 0.95 (95%CI: 0.76 ~ 0.99), a specificity of 0.95 (95%CI: 0.84 ~ 0.99), a PLR of 20.4 (95%CI: 5.9 ~ 71.1), an NLR of 0.06 (95%CI: 0.01 ~ 0.28), a DOR of 367 (95%CI: 58 ~ 2324), and an AUROC of 0.99 (95%CI: 0.33 ~ 1.00). (Figure S5 and S6).

### Type of validation

#### Cross-validation

Cross-validation methods were used in 14 of the 25 models, with a sensitivity of 0.96 (95%CI: 0.89 ~ 0.98), a specificity of 0.99 (95%CI: 0.93 ~ 1.00), a PLR of 96.8 (95%CI: 13.6 ~ 689.3), an NLR of 0.04 (95%CI: 0.02 ~ 0.11), a DOR of 2184 (95%CI: 143 ~ 33,329), and an AUROC of 0.99 (95%CI: 0.34 ~ 1.00). (Figure S7 and S8).

#### Test set/validation set

Among the 25 models, 11 models were validated in either the test set or the validation set, with a sensitivity of 0.93 (95%CI: 0.81 ~ 0.97), a specificity of 0.97 (95%CI: 0.88 ~ 0.99), a PLR of 29.4 (95%CI: 7.3 ~ 118.9), an NLR of 0.08 (95%CI: 0.03 ~ 0.22), a DOR of 386 (95%CI: 46 ~ 3269), and an AUROC of 0.98 (95%CI: 0.63 ~ 1.00). (Figure S9 and S10).

#### Multi-class classification tasks

**Fig. 5:**
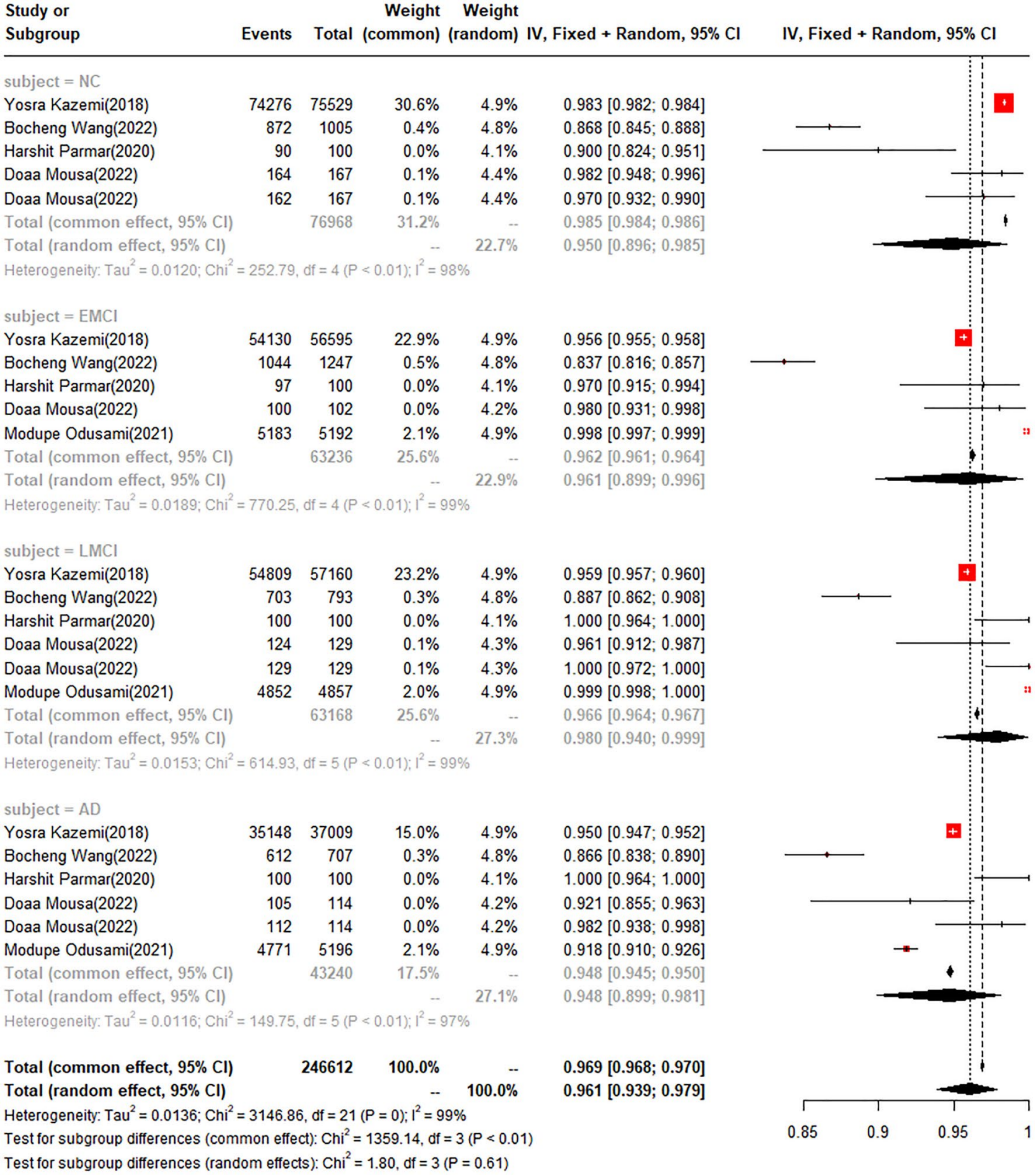
Forest plot for meta-analysis of total diagnostic accuracy for AD in multi-class classification tasks

In the multi-class classification tasks, the accuracy was 98.5% in the normal population (NC), 96.2% in the EMCI, 96.6% in LMCI, and 94.8% in AD. (Fig. 5).

## Discussion

### Summary of the main findings

Regarding the ML performance in binary classification tasks for AD diagnosis, this systematic review found that ML can effectively diagnose AD with a sensitivity of 0.91 and a specificity of 0.97. This demonstrates that the ML model can complete the disease diagnosis task based on Rs-fMRI.

The DL method had higher diagnostic accuracy compared to traditional ML methods with a sensitivity and specificity of 0.96 and 0.99, respectively, both of which were higher than that of ML model, suggesting that it has better performance in disease diagnosis.

The support vector machines (SVM) were found to be the most effective in predicting disease among the ML models, with a sensitivity of 0.95, which was higher compared with the average value of ML.

Further analysis revealed that the predictive accuracy of cross-validation methods was higher than that of the test set or validation set validation methods. Such methods exhibited good diagnostic accuracy for different populations (NC, EMCL, LMCL, AD) in multi-class classification tasks.

### Comparison with previous reviews

Neuroimaging techniques, including sMRI, fMRI, PET, and single photon emission computed tomography (SPECT) have been widely applied in AD diagnosis. These modalities are particularly valuable for early disease detection and differentiating AD subtypes. Among these, PET and sMRI are the most commonly utilized imaging techniques.

PET can detect pathological changes in the nervous system at an early stage to achieve early and accurate diagnosis of AD. PET can also provide information about the function, metabolism, and perfusion status of the nervous system, which can facilitate accurate determination of the severity of the disease, as well as the efficacy of treatments. Positron emission tomography with 2-deoxy-2-[fluorine-18] fluoro-D-glucose coupled with computed tomography (^18^F-FDG PET) has been used as an adjunct for AD diagnosis for more than 20 years. AD is characterized with low glucose metabolism in the temporoparietal and posterior cingulate regions [46]. Multicenter studies have reported that ^18^F-FDG PET correctly classifies 95% of AD patients, and can effectively predict the transition of MCI to AD [47]. In contrast, radiotracers that detect amyloid-beta (Aβ) accumulation, tau protein aggregation, and neuroinflammation more directly reflect the pathological state of individuals with AD or MCI progressing to AD (cMCI) [48]. While both are valuable, amyloid-beta (Aβ) imaging with radiotracers currently holds a more established clinical role compared to measures of gray matter atrophy and reduced cerebral glucose metabolism in predicting the progression from MCI to AD [49]. It is, therefore, valuable in the early diagnosis of AD. A previous meta-analysis comprising 5,967 patients explored the diagnostic value of Aβ-PET in AD [13], and reported a sensitivity, specificity, DOR, and AUC of 0.90, 0.80, 35.68, and 0.91, respectively. Subgroup analyses showed that Aβ-PET had a high sensitivity (0.91) and specificity (0.81) in distinguishing AD from normal controls. However, its specificity in differentiating AD from MCI was poor (0.49). Moreover, its sensitivity and specificity in predicting the transition from MCI to AD were 0.84 and 0.62, respectively. Compared with PET, Rs-fMRI had better performance in predicting the progression from MCI to AD.

sMRI can be applied in the detection and evaluation of neurodegenerative lesions in patients with AD owing to its ability to provide detailed visualization of brain structures [14]. For example, sMRI can diagnose AD by measuring the volume and morphology of the hippocampus, a reduction in hippocampus volume is an important feature of AD. In addition, sMRI can detect whole-brain atrophy associated with AD, especially in the medial temporal lobe [50]. However, limitations such as the high dimensionality of raw sMRI images and the lack of obvious structural changes in early AD may decrease its performance in early assessment and diagnosis of the disease. A previous review involving 3,935 participants explored the sensitivity and specificity of sMRI in diagnosing AD [14]. The volume of the total hippocampus was reported in most of the included literature with a pooled mean sensitivity of 0.73 (95% confidence interval (CI) 0.64 to 0.80) and a pooled mean specificity of 0.71 (95% CI 0.65 to 0.77). Atrophy of the medial temporal lobe was reported in some of the included studies with a mean sensitivity of 0.64 (95% CI 0.53 to 0.73) and a mean specificity of 0.65 (95% CI 0.51 to 0.76), involving 1,077 participants. Five studies reported the volume of lateral ventricles, demonstrating a mean sensitivity of 0.57 (95% CI: 0.49–0.65) and a mean specificity of 0.64 (95% CI: 0.59–0.70). Analysis of the most extensively studied brain regions, the hippocampus and medial temporal lobe, revealed low sensitivity and specificity for volumetry as an independent biomarker using sMRI for early AD diagnosis in individuals with MCI.

In this review, we found that Rs-fMRI provided a better diagnosis of early AD compared with CT and sMRI. In addition, Rs-fMRI was more effective in predicting the transition from MCI to AD compared with PET. The advantages of Rs-fMRI in diagnosing AD are detailed in Table 2. As stated in the introduction, measures for evaluating regional brain activity include rsFC, ReHo, and ALFF analysis, all of which can describe Rs-fMRI images. ReHo describes intra-regional coherence and rsFC describes synchronization between regions. Notably, none of them can directly describe the intensity of brain activity in a region, i.e., activity detection is not possible. ALFF reveals the intensity of the BOLD signal for spontaneous regional activity [51]. One study included in our analysis reported the use of fractional anisotropy (FC) as an evaluation indicator [40]. While current imaging techniques have limitations, ongoing technological advancements are expected to expand the application of brain imaging in AD diagnosis and treatment, leading to more effective therapeutic and preventive strategies.

**Table 2:** Comparison of neuroimaging techniques in the diagnosis of AD

Method	Identifying AD	Early identification	Identifying the ability of MCI to convert to AD
PET	+	+	-
sMRI	+	-	-
Rs-fMRI	+	+	+

The types of tasks in ML are grouped into two: binary classification and multi-class classification. The number of binary classification tasks in the included studies was much higher than that of multi-class classification tasks. However, in clinical practice, clinicians tend to focus more on multi-class classification tasks. This is because during differential diagnosis, AD should be differentiated from several other diseases with similar manifestations, and the stage of the disease and degree of impairment need to be clarified. This cannot be achieved using a simple binary classification task. Therefore, more complex multi-class classification tasks should be designed to enhance the clinical diagnosis of disease.

In this systematic review, data were obtained from included studies, eliminating the need for manual coding. Our analysis confirmed the superior diagnostic performance of DL compared to traditional methods. By intelligently extracting image data, DL can mitigate the variability inherent in manually defined regions of interest. Future research should focus on advancing DL theories to develop sophisticated image analysis tools.

### Advantages and limitations of the study

Rs-fMRI provide a non-invasive approach for assessing the functional brain connectivity with potential to detect early damage in AD. MCI occurs during the early stage of AD. In this systematic review, we identified the model types and validation types with high diagnostic accuracy for AD. Moreover, we examined binary and multi-class classification tasks separately and demonstrated the significance of multi-class classification in clinical setting. To minimize limitations associated with the heterogeneity among the studies, we performed subgroup analysis to compare the performance of DL and ML models.

Despite the strong diagnostic evidence mentioned above, this systematic review and meta-analysis has several limitations. One of them is the potential for sample bias due to the heavy reliance on the ADNI database, making it difficult to assess population overlap. The inclusion of only a limited number of case–control studies further restricted the analysis. Notably, two studies by Ali Khazaee et al. [40, 41] were included, both exploring deep learning and machine learning models. This overlap might have influenced the observed lower sensitivity of machine learning models compared to deep learning approaches.

Secondly, there was a potential publication bias in the patient-based analysis. Some unimportant or undesirable results are not always reported. However, such results may affect the diagnostic performance.

Thirdly, several clinical tools have been developed to facilitate the diagnosis of AD, but we only focused on the diagnostic performance of Rs-fMRI. For example, besides imaging methods, cerebrospinal fluid biomarkers are commonly employed in the diagnosis of AD. Unfortunately, none of the studies included in this study used cerebrospinal fluid biomarkers (e.g., CSF/ pet-amyloid biomarkers, etc.) for the diagnosis of AD. This may be due to the fact that CSF is collected using invasive procedures that are not often accepted by patients. There is Only one study reported the use of lumbar puncture to assess their CSF/pet -amyloid biomarker levels [29]. In the study, they compared the gap between CSF/pet -amyloid biomarkers and the predictive performance of RsfMRI. The patient inclusion criteria did not require CSF or PET amyloid biomarker assessment. Additionally, the use of the Clinical Dementia Rating Scale (CDR) and the Modified Mini-Mental State Examination (MMSE) for diagnosing MCI and AD introduced potential methodological biases. To enhance the generalizability and predictive accuracy of future models, larger and more diverse datasets incorporating amyloid biomarker data are warranted.

Fourthly, each type of MCI has a unique etiology. Various factors have been reported including vascular disease, Parkinson's disease (PD), dementia with Lewy bodies (DLB), frontotemporal lobe dementia (FTLD), long-term sleep deprivation, and heavy metal poisoning. Therefore, the progression of MCI to AD may be influenced by several risk factors such as hypertension, diabetes, obesity, early malnutrition, hearing loss, smoking, and alcohol consumption. The studies enrolled in our study did not differentiate the different causes of MCI. The absence of amyloid biomarkers to definitively confirm AD etiology may have introduced bias in diagnosing MCI, potentially affecting the performance of ML and DL models.

Finally, our study adopted a single-modality approach, relying solely on Rs-fMRI data. While we acknowledge the potential benefits of multimodal imaging, particularly in enhancing diagnostic accuracy and classification, for this study, we focused on exploring the capabilities of rs-fMRI. Other research has demonstrated the value of multimodal approaches, such as combining resting-state FDG-PET with fMRI, to investigate the relationship between brain glucose metabolism and activity in AD patients [52]. In addition, a previous study demonstrated that hippocampal subregion and amygdala volumes obtained by sMRI in combination with brain network features and multiple measurement features from Rs-fMRI could effectively achieve early diagnosis and classification of AD [35]. A multimodal approach using a combination of diagnostic tools can provide higher diagnostic accuracy, facilitate early diagnosis, reveal the pathological mechanisms, and reduce misdiagnosis rates. Therefore, we will also explore the possibility of combining Rs-fMRI data with other biomarkers in subsequent studies.

## Conclusions

In summary, several studies have explored various techniques for diagnosing MCI and AD in recent years. With the development of brain imaging technology, several tests have been established. Among them, Rs-fMRI has attracted attention because of its non-invasive nature and its ability to recognize early development in AD. The emergence of AI tools has revolutionized various aspects of AD detection and management. For underdeveloped regions where medical resources are relatively scarce, early identification of AD can be achieved using AI methods, which will allow establishment of a better prognosis.

The results of this review demonstrate that Rs-fMRI can diagnosis AD and its accuracy can be improved through AI methods. However, there are some limitations of this study. Therefore, future prospective, multicenter studies are advocated to explore the potential for application of this technique in routine AD diagnosis.

## Supplementary Information

Supplementary Material 1: Figure S1 Forest plot of meta-analysis of AD diagnostic accuracy for DL models in binary classification tasks. Figure S2 SROC curves for meta-analysis of AD diagnostic accuracy for DL models in binary classification tasks. Figure S3 Forest plot of meta-analysis of AD diagnostic accuracy for ML models in binary classification tasks. Figure S4 SROC curves for meta-analysis of AD diagnostic accuracy for ML models in binary classification tasks. Figure S5 Forest plot of meta-analysis of AD diagnostic accuracy for SVM models in binary classification tasks. Figure S6 SROC curves for meta-analysis of AD diagnostic accuracy in SVM models in binary classification tasks. Figure S7 Forest plot of meta-analysis of AD diagnostic accuracy for cross-validation in binary classification tasks. Figure S8 SROC curves for meta-analysis of AD diagnostic accuracy for cross-validation in binary classification tasks. Figure S9 Forest plot of meta-analysis of AD diagnostic accuracy for the test set/verification set in binary classification tasks. Figure S10 SROC curves for meta-analysis of AD diagnostic accuracy for the test set/verification set in binary classification tasks. Table S1 Illustration of the search strategy. Table S2 Quality Assessment of Diagnostic Accuracy Studies

## Data Availability

The data that support the findings of this study are available from the corresponding author upon reasonable request.

## Abbreviations

ML: Machine learning
Rs-fMRI: Resting-state functional MRI
MCI: Mild cognitive impairment
AD: Alzheimer's disease
EMCI: Early mild cognitive impairment
LMCI: Late mild cognitive impairment
CT: Computed tomography
PET: Positron emission tomography
sMRI: Structural magnetic resonance imaging
Rs-fMRI: Resting-state functional magnetic resonance imaging
BOLD: Blood-oxygen level-dependent
ReHo: Regional homogeneity
ALFF: Amplitude of Low Frequency Fluctuation
rsFC: Resting state functional connectivity
AI: Artificial intelligence
ML: Machine learning
DL: Deep learning
The PRISMA-DTA Statement: The Preferred Reporting Items for a Systematic Review and Meta-analysis of Diagnostic Test Accuracy Studies
RQS: Radiomics Quality Score
PLR: Positive likelihood ratio
NLR: Negative likelihood ratio
DOR: Diagnostic odds ratio
SROC: Summary ROC curve
QUADAS-2: Quality Assessment of Diagnostic Accuracy Studies-2
CDR: Clinical Dementia Rating Scale
MMSE: Modified Mental State Examination
SVM: Support vector machine
NC: Normal population
SPECT: Single photon emission computed tomography
Aβ: Amyloid-β
cMCI: Converting to AD
CI: Confidence interval
