# Supplementary material for: The application value of Rs-fMRI-based machine learning models for differentiating mild cognitive impairment from Alzheimer's disease: a systematic review and meta-analysis
Source: Neurol Sci. 2024 Sep 3;46(1):45–62. doi: 10.1007/s10072-024-07731-1 (PMC11698789; doi:10.1007/s10072-024-07731-1)
Supplement: Supplementary file 1 — Supplementary Material 1: Figure S1 Forest plot of meta-analysis of AD diagnostic accuracy for DL models in binary classification tasks. Figure S2 SROC curves for meta-analysis of AD diagnostic accuracy for DL models in binary classification tasks. Figure S3 Forest plot of meta-analysis of AD diagnostic accuracy for ML models in binary classification tasks. Figure S4 SROC curves for meta-analysis of AD diagnostic accuracy for ML models in binary classification tasks. Figure S5 Forest plot of meta-analysis of AD diagnostic accuracy for SVM models in binary classification tasks. Figure S6 SROC curves for meta-analysis of AD diagnostic accuracy in SVM models in binary classification tasks. Figure S7 Forest plot of meta-analysis of AD diagnostic accuracy for cross-validation in binary classification tasks. Figure S8 SROC curves for meta-analysis of AD diagnostic accuracy for cross-validation in binary classification tasks. Figure S9 Forest plot of meta-analysis of AD diagnostic accuracy for the test set/verification set in binary classification tasks. Figure S10 SROC curves for meta-analysis of AD diagnostic accuracy for the test set/verification set in binary classification tasks. Table S1 Illustration of the search strategy. Table S2 Quality Assessment of Diagnostic Accuracy Studies [file 10072_2024_7731_MOESM1_ESM.docx]

**Supplementary Materials**

Figure S1 Forest plot of meta-analysis of AD diagnostic accuracy for DL models in binary classification tasks

Figure S2 SROC curves for meta-analysis of AD diagnostic accuracy for DL models in binary classification tasks

Figure S3 Forest plot of meta-analysis of AD diagnostic accuracy for ML models in binary classification tasks

Figure S4 SROC curves for meta-analysis of AD diagnostic accuracy for ML models in binary classification tasks

Figure S5 Forest plot of meta-analysis of AD diagnostic accuracy for SVM models in binary classification tasks

Figure S6 SROC curves for meta-analysis of AD diagnostic accuracy in SVM models in binary classification tasks

Figure S7 Forest plot of meta-analysis of AD diagnostic accuracy for cross-validation in binary classification tasks

Figure S8 SROC curves for meta-analysis of AD diagnostic accuracy for cross-validation in binary classification tasks

Figure S9 Forest plot of meta-analysis of AD diagnostic accuracy for the test set/verification set in binary classification tasks

Figure S10 SROC curves for meta-analysis of AD diagnostic accuracy for the test set/verification set in binary classification tasks

Table S1 Search strategy

Table S2 Quality Assessment of Diagnostic Accuracy Studies

**
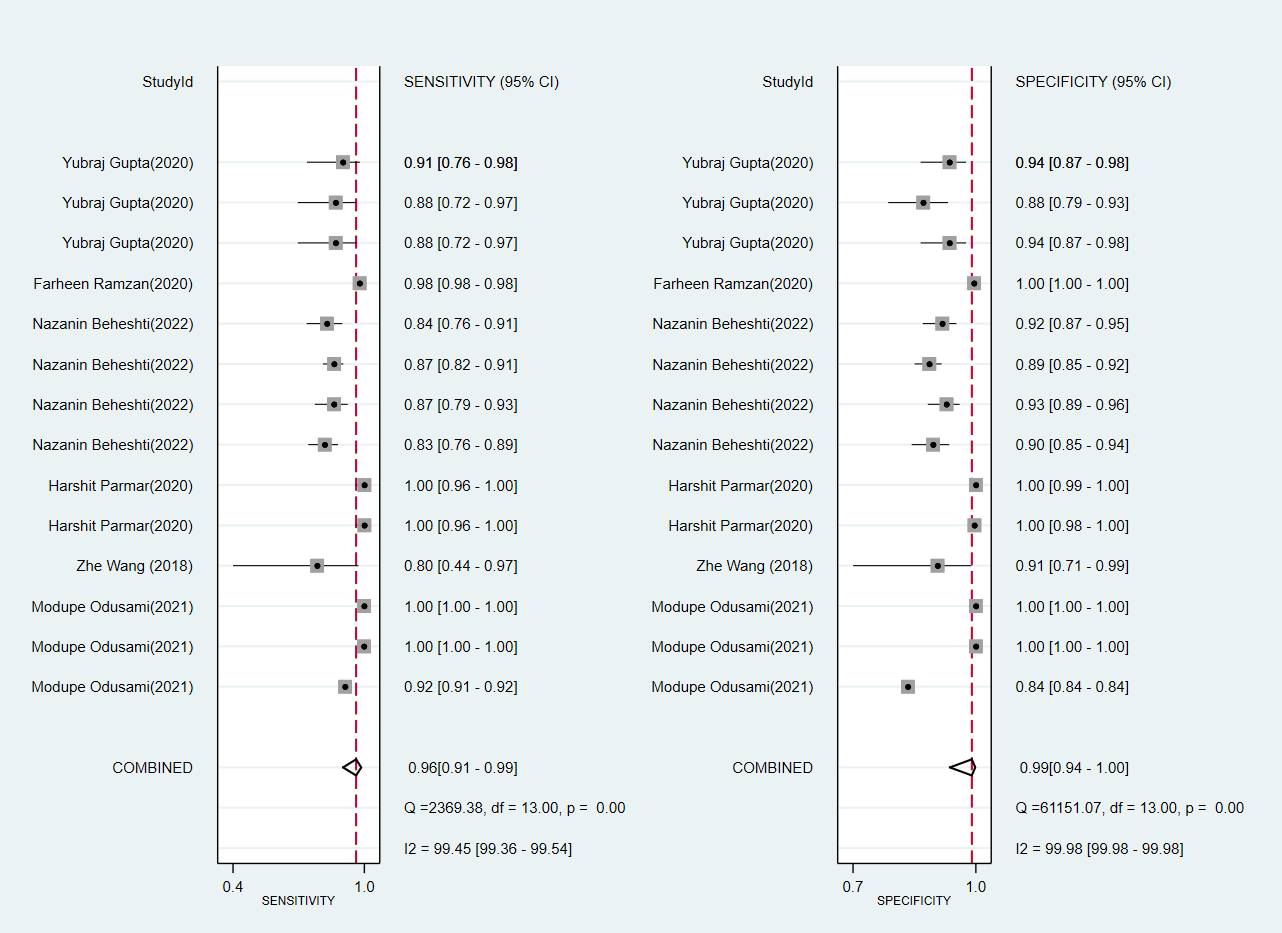
**

**Figure S1**. Forest plot of meta-analysis of AD diagnostic accuracy for DL models in binary classification tasks


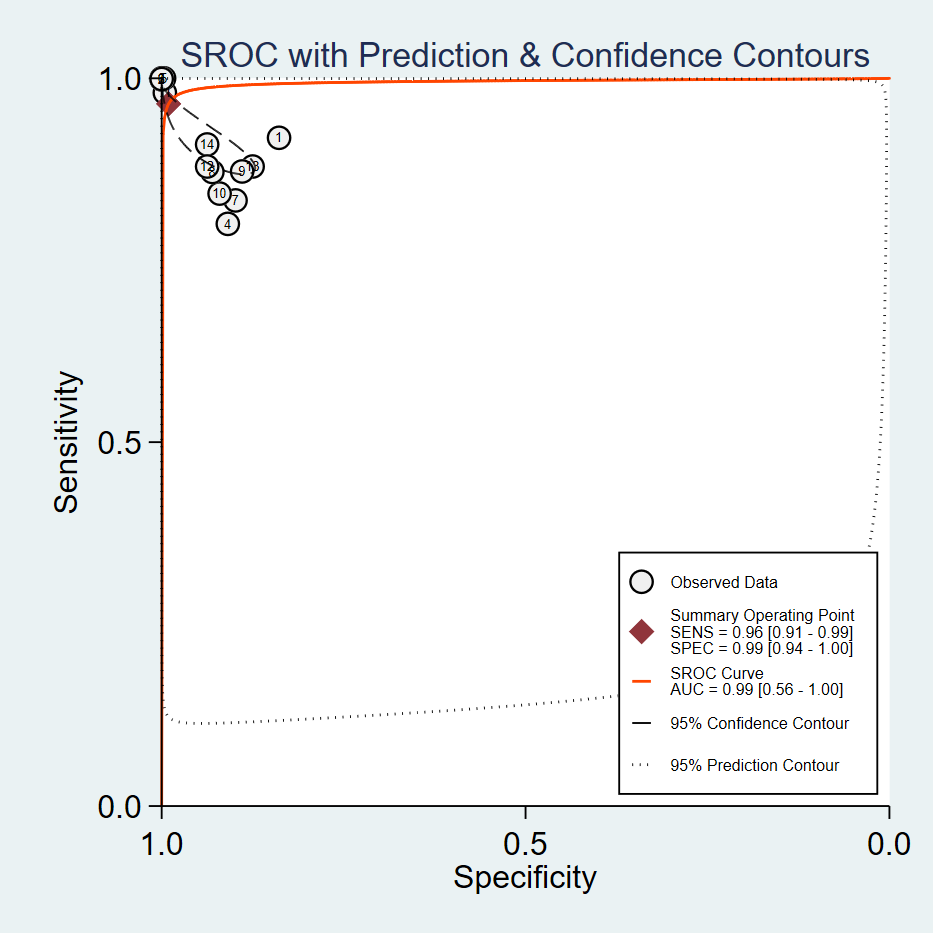


**Figure S2**. SROC curves for meta-analysis of AD diagnostic accuracy for DL models in binary classification tasks


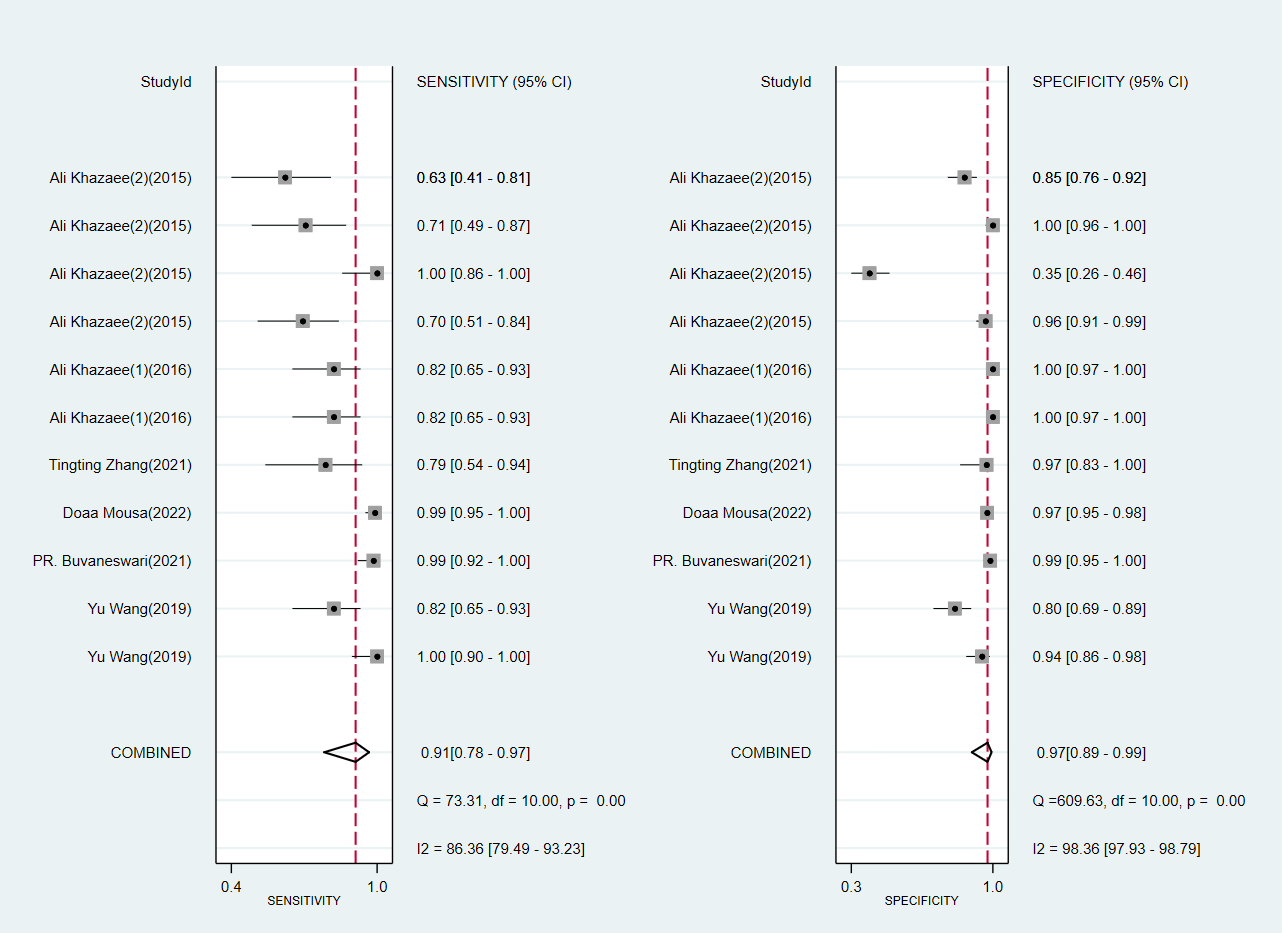


**Figure S3**. Forest plot of meta-analysis of AD diagnostic accuracy for ML models in binary classification tasks


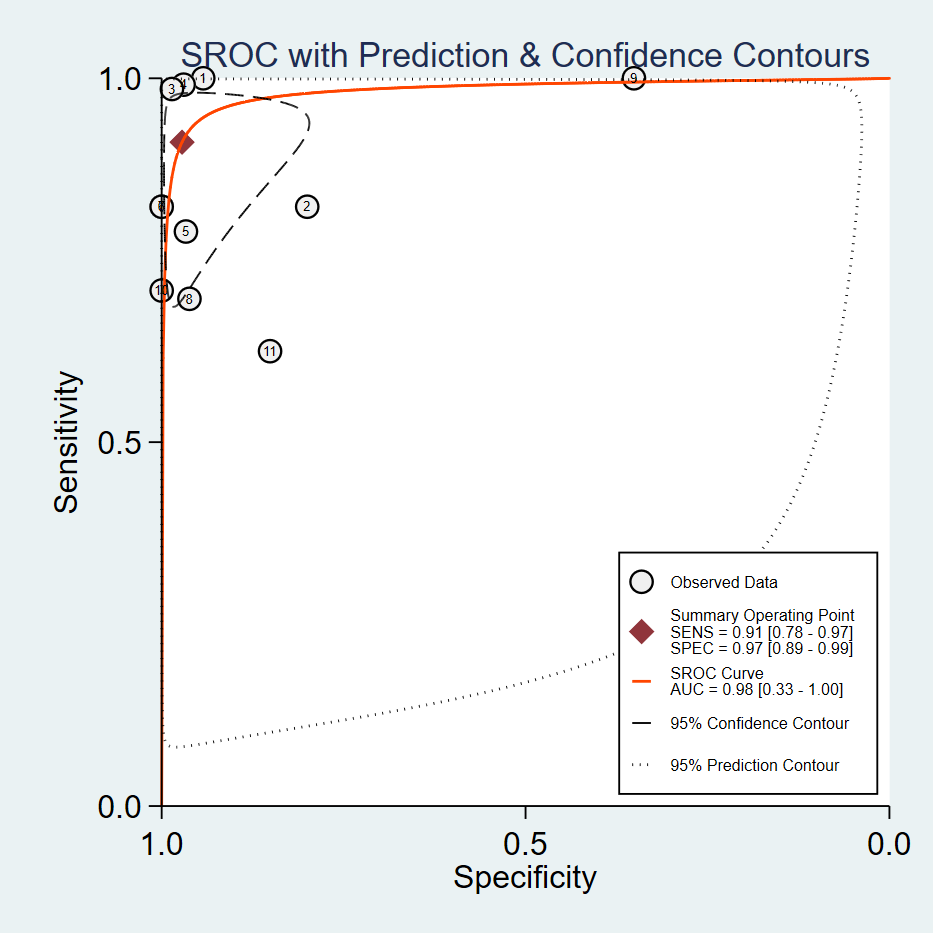


**Figure S4**. SROC curves for meta-analysis of AD diagnostic accuracy for ML models in binary classification tasks


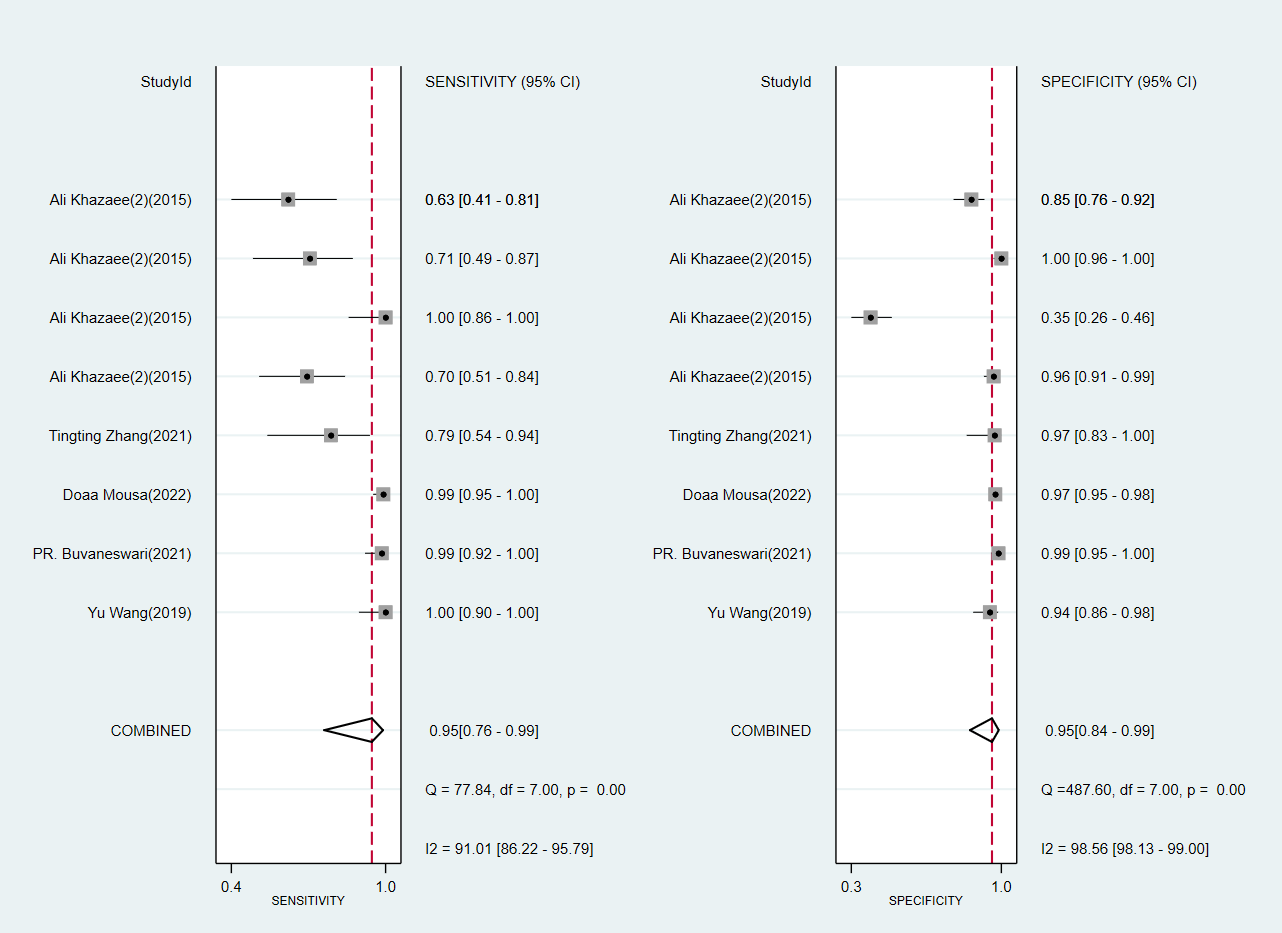


**Figure S5**. Forest plot of meta-analysis of AD diagnostic accuracy for SVM models in binary classification tasks


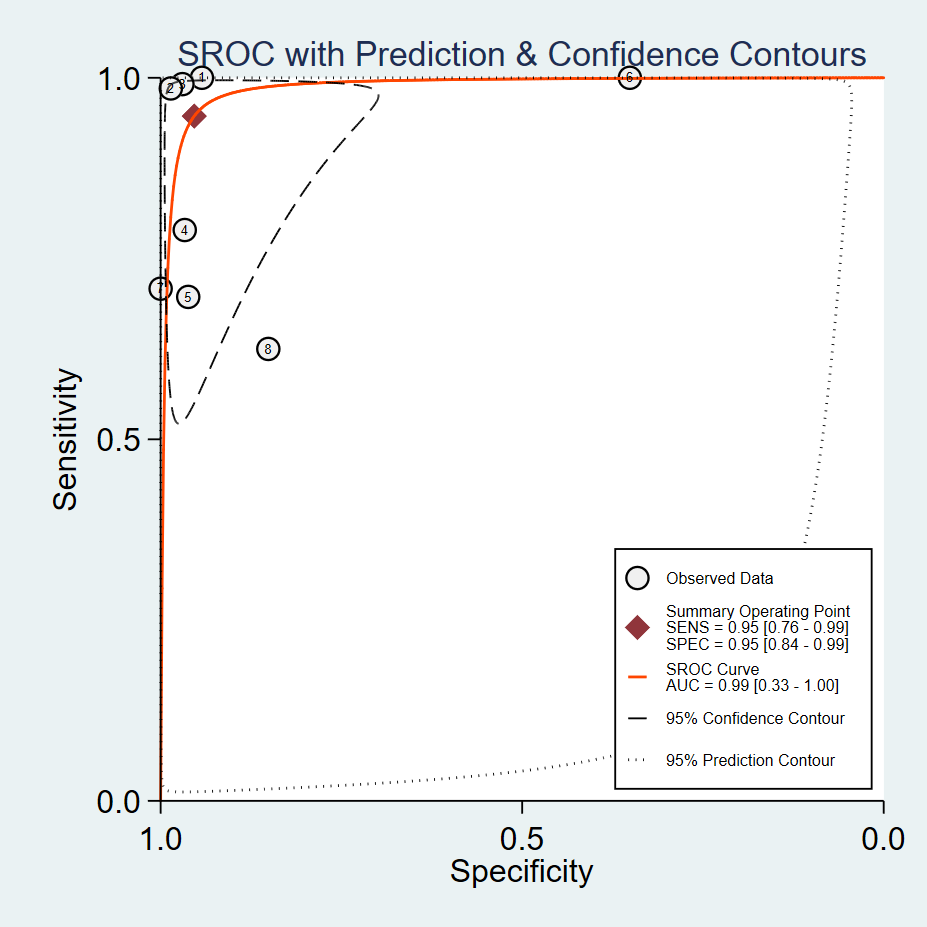


**Figure S6**. SROC curves for meta-analysis of AD diagnostic accuracy in SVM models in binary classification tasks


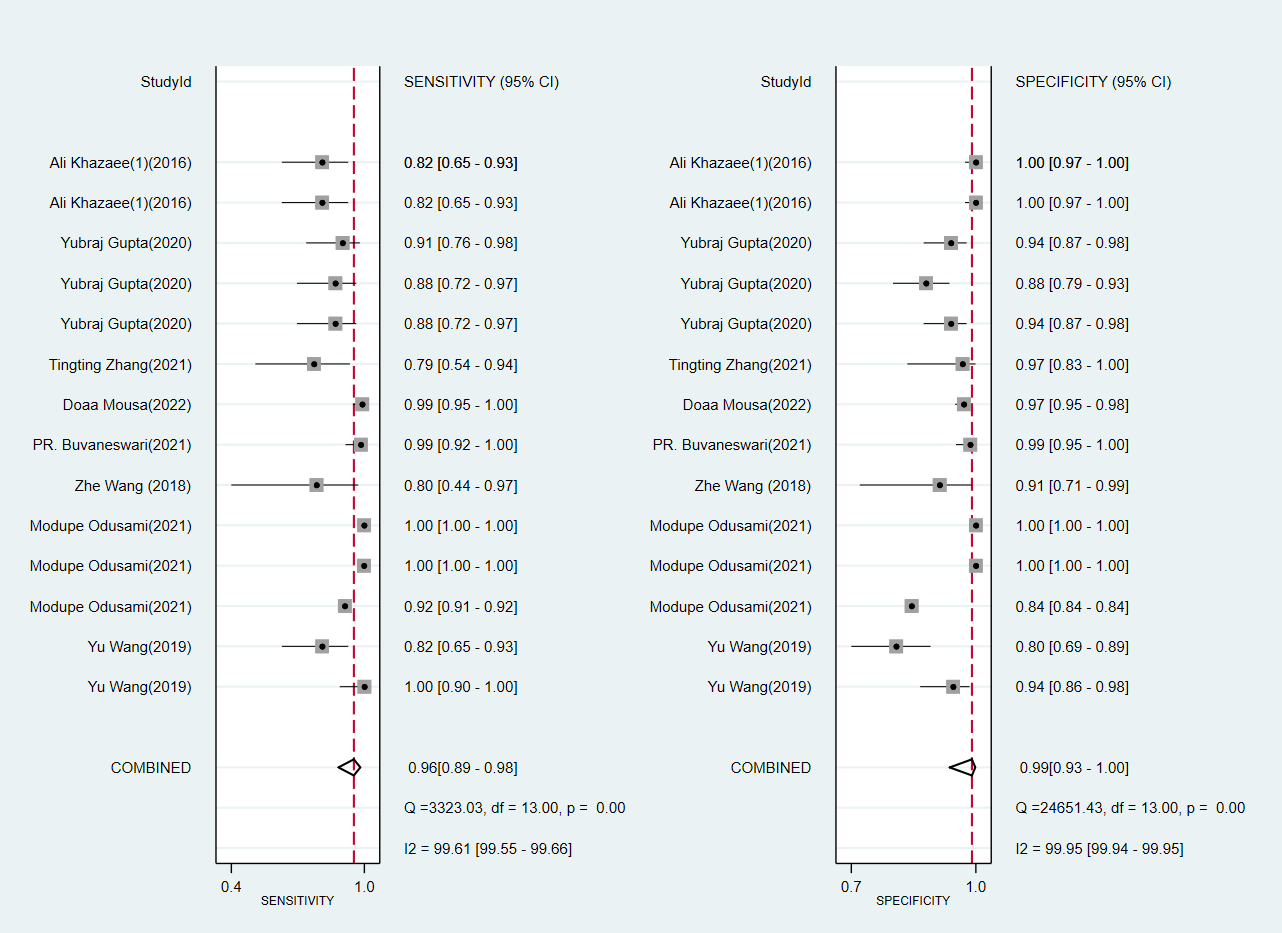


**Figure S7**. Forest plot of meta-analysis of AD diagnostic accuracy for cross-validation in binary classification tasks


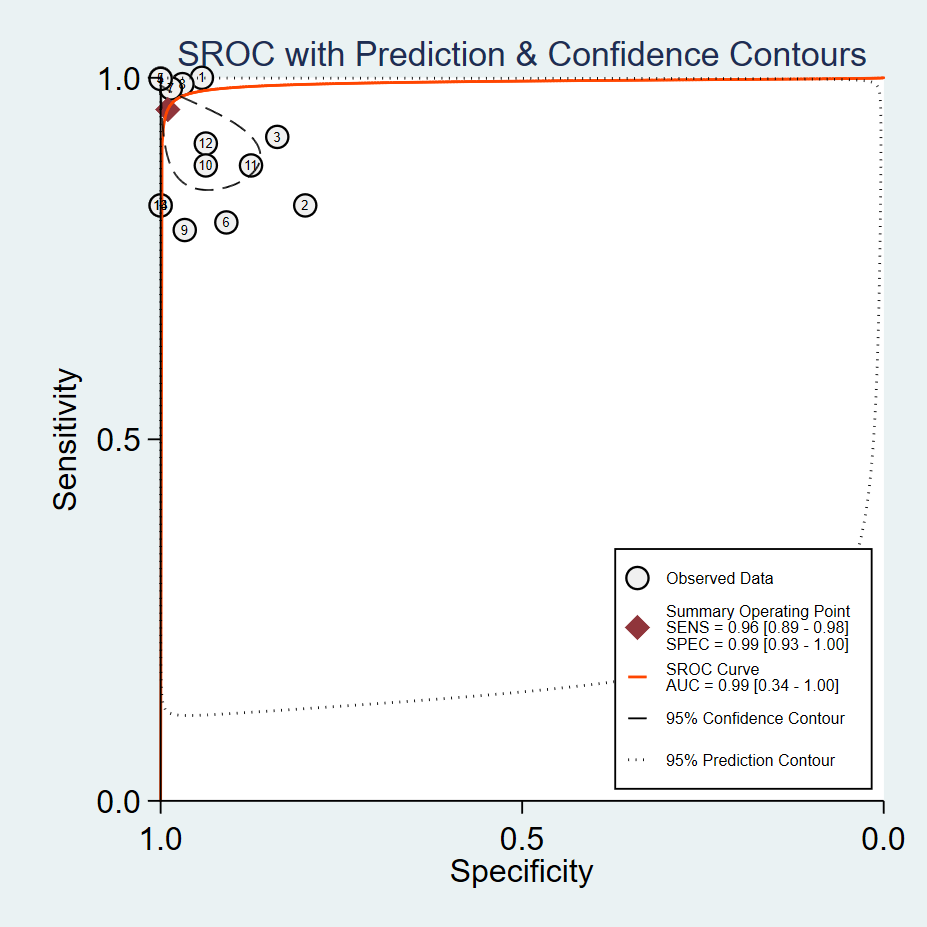


**Figure S8**. SROC curves for meta-analysis of AD diagnostic accuracy for cross-validation in binary classification tasks


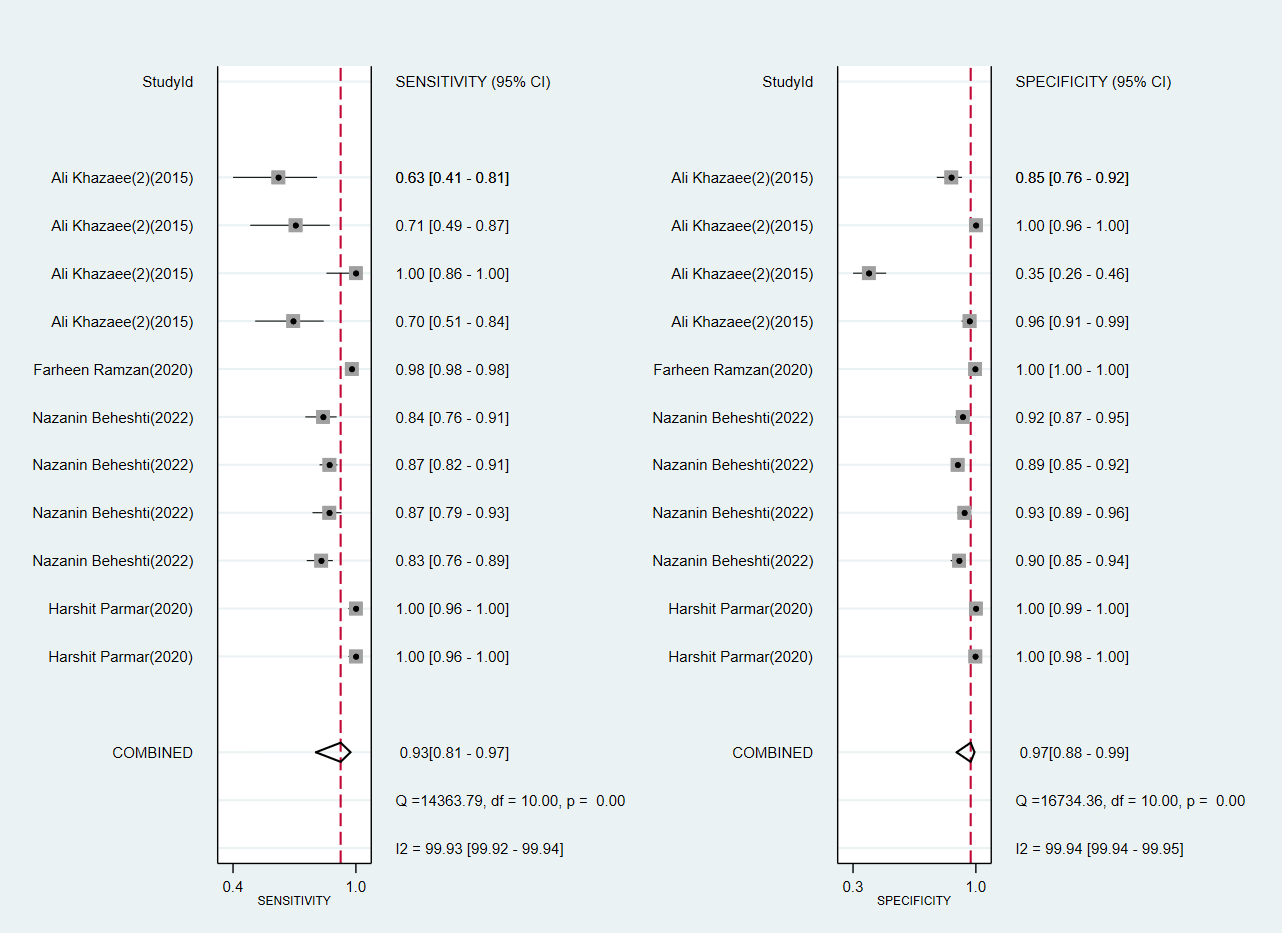


**Figure S9**. Forest plot of meta-analysis of AD diagnostic accuracy for the test set/verification set in binary classification tasks


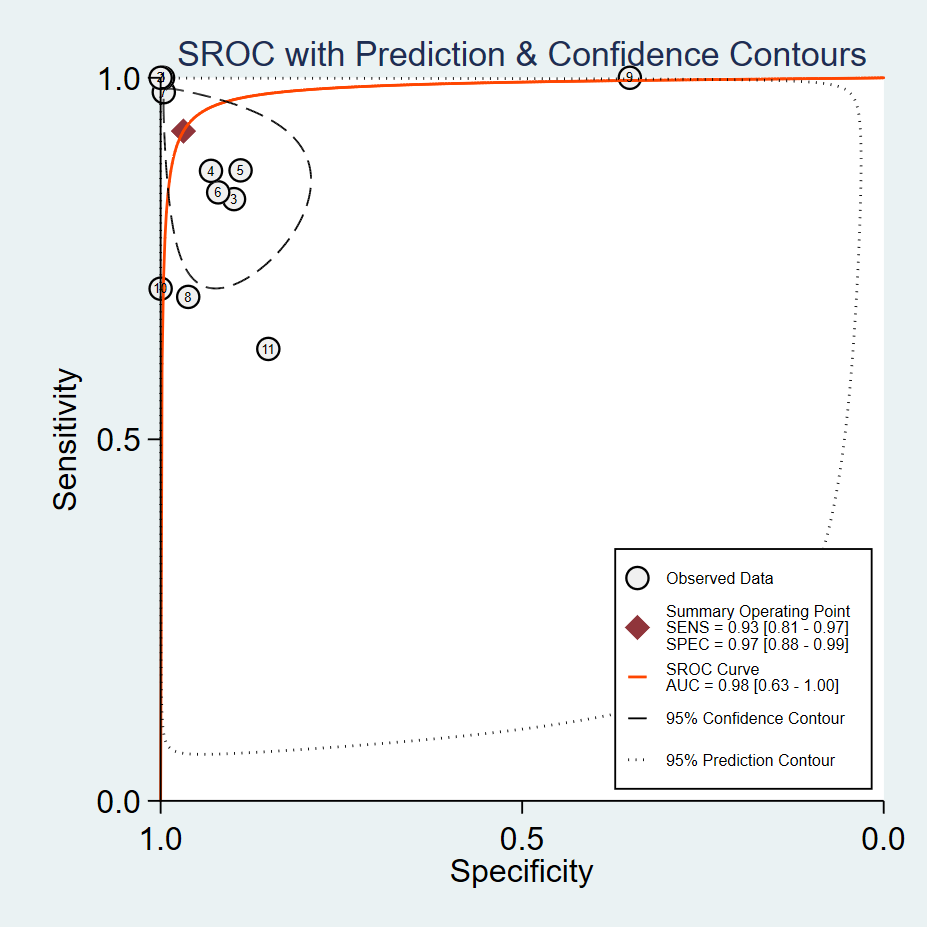


**Figure S10**. SROC curves for meta-analysis of AD diagnostic accuracy for the test set/verification set in binary classification tasks

**Table S1 Search strategy**

**1.Pubmed 1954**

| Search number | Query | Sort By | Filters | Search Details | Results | Time |
| --- | --- | --- | --- | --- | --- | --- |
| 10 | ((("Machine Learning"[Mesh]) OR ((((((((((((((((((((((((((machine learning[Title/Abstract]) OR (Transfer Learning[Title/Abstract])) OR (Deep learning[Title/Abstract])) OR (Ensemble Learning[Title/Abstract])) OR (artificial intelligence[Title/Abstract])) OR (Prediction model[Title/Abstract])) OR (Prediction models[Title/Abstract])) OR (random forest[Title/Abstract])) OR (neural network[Title/Abstract])) OR (neural networks[Title/Abstract])) OR (CNN[Title/Abstract])) OR (Support vector machine[Title/Abstract])) OR (SVM[Title/Abstract])) OR (Gradient Boosting Machine[Title/Abstract])) OR (GBM[Title/Abstract])) OR (Nomogram[Title/Abstract])) OR (XGBoost[Title/Abstract])) OR (Adaboost[Title/Abstract])) OR (Decision tree[Title/Abstract])) OR (ResNet-50[Title/Abstract])) OR (ResNet[Title/Abstract])) OR (Radiomics[Title/Abstract])) OR (Radiomic[Title/Abstract])) OR (Naive Bayesian[Title/Abstract])) OR (Risk Prediction[Title/Abstract])) OR (Risk-Prediction[Title/Abstract]))) AND (("Cognitive Dysfunction"[Mesh]) OR ((((((((((Cognitive Dysfunction[Title/Abstract]) OR (Cognitive Dysfunctions[Title/Abstract])) OR (Cognitive Impairments[Title/Abstract])) OR (Cognitive Impairment[Title/Abstract])) OR (Cognitive Disorder[Title/Abstract])) OR (Cognitive Disorders[Title/Abstract])) OR (Cognitive Decline[Title/Abstract])) OR (Cognitive Declines[Title/Abstract])) OR (Mental Deterioration[Title/Abstract])) OR (Mental Deteriorations[Title/Abstract])))) AND (("Alzheimer Disease"[Mesh]) OR (((((((((((((Alzheimer Disease[Title/Abstract]) OR (Alzheimer Dementia[Title/Abstract])) OR (Alzheimer Dementias[Title/Abstract])) OR (Alzheimer's Disease[Title/Abstract])) OR (Senile Dementia[Title/Abstract])) OR (Alzheimer Type Dementia[Title/Abstract])) OR (Primary Senile Degenerative Dementia[Title/Abstract])) OR (Alzheimer Sclerosis[Title/Abstract])) OR (Alzheimer Syndrome[Title/Abstract])) OR (Alzheimer's Diseases[Title/Abstract])) OR (Alzheimer Diseases[Title/Abstract])) OR (Alzheimers Diseases[Title/Abstract])) OR (Presenile Dementia[Title/Abstract]))) |  |  | ("Machine Learning"[MeSH Terms] OR ("Machine Learning"[Title/Abstract] OR "transfer learning"[Title/Abstract] OR "deep learning"[Title/Abstract] OR "ensemble learning"[Title/Abstract] OR "artificial intelligence"[Title/Abstract] OR "prediction model"[Title/Abstract] OR "prediction models"[Title/Abstract] OR "random forest"[Title/Abstract] OR "neural network"[Title/Abstract] OR "neural networks"[Title/Abstract] OR "CNN"[Title/Abstract] OR "support vector machine"[Title/Abstract] OR "SVM"[Title/Abstract] OR "gradient boosting machine"[Title/Abstract] OR "GBM"[Title/Abstract] OR "Nomogram"[Title/Abstract] OR "XGBoost"[Title/Abstract] OR "Adaboost"[Title/Abstract] OR "decision tree"[Title/Abstract] OR "ResNet-50"[Title/Abstract] OR "ResNet"[Title/Abstract] OR "Radiomics"[Title/Abstract] OR "Radiomic"[Title/Abstract] OR "naive bayesian"[Title/Abstract] OR "Risk-Prediction"[Title/Abstract] OR "Risk-Prediction"[Title/Abstract])) AND ("Cognitive Dysfunction"[MeSH Terms] OR ("Cognitive Dysfunction"[Title/Abstract] OR "cognitive dysfunctions"[Title/Abstract] OR "cognitive impairments"[Title/Abstract] OR "cognitive impairment"[Title/Abstract] OR "cognitive disorder"[Title/Abstract] OR "cognitive disorders"[Title/Abstract] OR "cognitive decline"[Title/Abstract] OR "cognitive declines"[Title/Abstract] OR "mental deterioration"[Title/Abstract] OR "mental deteriorations"[Title/Abstract])) AND ("Alzheimer Disease"[MeSH Terms] OR ("Alzheimer Disease"[Title/Abstract] OR "alzheimer dementia"[Title/Abstract] OR "alzheimer dementias"[Title/Abstract] OR "alzheimer s disease"[Title/Abstract] OR "senile dementia"[Title/Abstract] OR "alzheimer type dementia"[Title/Abstract] OR "primary senile degenerative dementia"[Title/Abstract] OR "alzheimer sclerosis"[Title/Abstract] OR "alzheimer syndrome"[Title/Abstract] OR "alzheimer s diseases"[Title/Abstract] OR "alzheimer diseases"[Title/Abstract] OR "alzheimers diseases"[Title/Abstract] OR "presenile dementia"[Title/Abstract])) | 1,954 | 2:43:03 |
| 9 | ("Alzheimer Disease"[Mesh]) OR (((((((((((((Alzheimer Disease[Title/Abstract]) OR (Alzheimer Dementia[Title/Abstract])) OR (Alzheimer Dementias[Title/Abstract])) OR (Alzheimer's Disease[Title/Abstract])) OR (Senile Dementia[Title/Abstract])) OR (Alzheimer Type Dementia[Title/Abstract])) OR (Primary Senile Degenerative Dementia[Title/Abstract])) OR (Alzheimer Sclerosis[Title/Abstract])) OR (Alzheimer Syndrome[Title/Abstract])) OR (Alzheimer's Diseases[Title/Abstract])) OR (Alzheimer Diseases[Title/Abstract])) OR (Alzheimers Diseases[Title/Abstract])) OR (Presenile Dementia[Title/Abstract])) |  |  | "Alzheimer Disease"[MeSH Terms] OR "Alzheimer Disease"[Title/Abstract] OR "alzheimer dementia"[Title/Abstract] OR "alzheimer dementias"[Title/Abstract] OR "alzheimer s disease"[Title/Abstract] OR "senile dementia"[Title/Abstract] OR "alzheimer type dementia"[Title/Abstract] OR "primary senile degenerative dementia"[Title/Abstract] OR "alzheimer sclerosis"[Title/Abstract] OR "alzheimer syndrome"[Title/Abstract] OR "alzheimer s diseases"[Title/Abstract] OR "alzheimer diseases"[Title/Abstract] OR "alzheimers diseases"[Title/Abstract] OR "presenile dementia"[Title/Abstract] | 200,877 | 2:42:30 |
| 8 | ((((((((((((Alzheimer Disease[Title/Abstract]) OR (Alzheimer Dementia[Title/Abstract])) OR (Alzheimer Dementias[Title/Abstract])) OR (Alzheimer's Disease[Title/Abstract])) OR (Senile Dementia[Title/Abstract])) OR (Alzheimer Type Dementia[Title/Abstract])) OR (Primary Senile Degenerative Dementia[Title/Abstract])) OR (Alzheimer Sclerosis[Title/Abstract])) OR (Alzheimer Syndrome[Title/Abstract])) OR (Alzheimer's Diseases[Title/Abstract])) OR (Alzheimer Diseases[Title/Abstract])) OR (Alzheimers Diseases[Title/Abstract])) OR (Presenile Dementia[Title/Abstract]) |  |  | "alzheimer disease"[Title/Abstract] OR "alzheimer dementia"[Title/Abstract] OR "alzheimer dementias"[Title/Abstract] OR "alzheimer s disease"[Title/Abstract] OR "senile dementia"[Title/Abstract] OR "alzheimer type dementia"[Title/Abstract] OR "primary senile degenerative dementia"[Title/Abstract] OR "alzheimer sclerosis"[Title/Abstract] OR "alzheimer syndrome"[Title/Abstract] OR "alzheimer s diseases"[Title/Abstract] OR "alzheimer diseases"[Title/Abstract] OR "alzheimers diseases"[Title/Abstract] OR "presenile dementia"[Title/Abstract] | 182,868 | 2:42:24 |
| 7 | "Alzheimer Disease"[Mesh] | Most Recent |  | "Alzheimer Disease"[MeSH Terms] | 122,934 | 2:41:01 |
| 6 | ("Cognitive Dysfunction"[Mesh]) OR ((((((((((Cognitive Dysfunction[Title/Abstract]) OR (Cognitive Dysfunctions[Title/Abstract])) OR (Cognitive Impairments[Title/Abstract])) OR (Cognitive Impairment[Title/Abstract])) OR (Cognitive Disorder[Title/Abstract])) OR (Cognitive Disorders[Title/Abstract])) OR (Cognitive Decline[Title/Abstract])) OR (Cognitive Declines[Title/Abstract])) OR (Mental Deterioration[Title/Abstract])) OR (Mental Deteriorations[Title/Abstract])) |  |  | "Cognitive Dysfunction"[MeSH Terms] OR "Cognitive Dysfunction"[Title/Abstract] OR "cognitive dysfunctions"[Title/Abstract] OR "cognitive impairments"[Title/Abstract] OR "cognitive impairment"[Title/Abstract] OR "cognitive disorder"[Title/Abstract] OR "cognitive disorders"[Title/Abstract] OR "cognitive decline"[Title/Abstract] OR "cognitive declines"[Title/Abstract] OR "mental deterioration"[Title/Abstract] OR "mental deteriorations"[Title/Abstract] | 146,588 | 2:40:35 |
| 5 | (((((((((Cognitive Dysfunction[Title/Abstract]) OR (Cognitive Dysfunctions[Title/Abstract])) OR (Cognitive Impairments[Title/Abstract])) OR (Cognitive Impairment[Title/Abstract])) OR (Cognitive Disorder[Title/Abstract])) OR (Cognitive Disorders[Title/Abstract])) OR (Cognitive Decline[Title/Abstract])) OR (Cognitive Declines[Title/Abstract])) OR (Mental Deterioration[Title/Abstract])) OR (Mental Deteriorations[Title/Abstract]) |  |  | "cognitive dysfunction"[Title/Abstract] OR "cognitive dysfunctions"[Title/Abstract] OR "cognitive impairments"[Title/Abstract] OR "cognitive impairment"[Title/Abstract] OR "cognitive disorder"[Title/Abstract] OR "cognitive disorders"[Title/Abstract] OR "cognitive decline"[Title/Abstract] OR "cognitive declines"[Title/Abstract] OR "mental deterioration"[Title/Abstract] OR "mental deteriorations"[Title/Abstract] | 140,452 | 2:40:26 |
| 4 | "Cognitive Dysfunction"[Mesh] | Most Recent |  | "Cognitive Dysfunction"[MeSH Terms] | 38,983 | 2:39:09 |
| 3 | ("Machine Learning"[Mesh]) OR ((((((((((((((((((((((((((machine learning[Title/Abstract]) OR (Transfer Learning[Title/Abstract])) OR (Deep learning[Title/Abstract])) OR (Ensemble Learning[Title/Abstract])) OR (artificial intelligence[Title/Abstract])) OR (Prediction model[Title/Abstract])) OR (Prediction models[Title/Abstract])) OR (random forest[Title/Abstract])) OR (neural network[Title/Abstract])) OR (neural networks[Title/Abstract])) OR (CNN[Title/Abstract])) OR (Support vector machine[Title/Abstract])) OR (SVM[Title/Abstract])) OR (Gradient Boosting Machine[Title/Abstract])) OR (GBM[Title/Abstract])) OR (Nomogram[Title/Abstract])) OR (XGBoost[Title/Abstract])) OR (Adaboost[Title/Abstract])) OR (Decision tree[Title/Abstract])) OR (ResNet-50[Title/Abstract])) OR (ResNet[Title/Abstract])) OR (Radiomics[Title/Abstract])) OR (Radiomic[Title/Abstract])) OR (Naive Bayesian[Title/Abstract])) OR (Risk Prediction[Title/Abstract])) OR (Risk-Prediction[Title/Abstract])) |  |  | "Machine Learning"[MeSH Terms] OR "Machine Learning"[Title/Abstract] OR "transfer learning"[Title/Abstract] OR "deep learning"[Title/Abstract] OR "ensemble learning"[Title/Abstract] OR "artificial intelligence"[Title/Abstract] OR "prediction model"[Title/Abstract] OR "prediction models"[Title/Abstract] OR "random forest"[Title/Abstract] OR "neural network"[Title/Abstract] OR "neural networks"[Title/Abstract] OR "CNN"[Title/Abstract] OR "support vector machine"[Title/Abstract] OR "SVM"[Title/Abstract] OR "gradient boosting machine"[Title/Abstract] OR "GBM"[Title/Abstract] OR "Nomogram"[Title/Abstract] OR "XGBoost"[Title/Abstract] OR "Adaboost"[Title/Abstract] OR "decision tree"[Title/Abstract] OR "ResNet-50"[Title/Abstract] OR "ResNet"[Title/Abstract] OR "Radiomics"[Title/Abstract] OR "Radiomic"[Title/Abstract] OR "naive bayesian"[Title/Abstract] OR "Risk-Prediction"[Title/Abstract] OR "Risk-Prediction"[Title/Abstract] | 365,208 | 2:35:53 |
| 2 | (((((((((((((((((((((((((machine learning[Title/Abstract]) OR (Transfer Learning[Title/Abstract])) OR (Deep learning[Title/Abstract])) OR (Ensemble Learning[Title/Abstract])) OR (artificial intelligence[Title/Abstract])) OR (Prediction model[Title/Abstract])) OR (Prediction models[Title/Abstract])) OR (random forest[Title/Abstract])) OR (neural network[Title/Abstract])) OR (neural networks[Title/Abstract])) OR (CNN[Title/Abstract])) OR (Support vector machine[Title/Abstract])) OR (SVM[Title/Abstract])) OR (Gradient Boosting Machine[Title/Abstract])) OR (GBM[Title/Abstract])) OR (Nomogram[Title/Abstract])) OR (XGBoost[Title/Abstract])) OR (Adaboost[Title/Abstract])) OR (Decision tree[Title/Abstract])) OR (ResNet-50[Title/Abstract])) OR (ResNet[Title/Abstract])) OR (Radiomics[Title/Abstract])) OR (Radiomic[Title/Abstract])) OR (Naive Bayesian[Title/Abstract])) OR (Risk Prediction[Title/Abstract])) OR (Risk-Prediction[Title/Abstract]) |  |  | "machine learning"[Title/Abstract] OR "transfer learning"[Title/Abstract] OR "deep learning"[Title/Abstract] OR "ensemble learning"[Title/Abstract] OR "artificial intelligence"[Title/Abstract] OR "prediction model"[Title/Abstract] OR "prediction models"[Title/Abstract] OR "random forest"[Title/Abstract] OR "neural network"[Title/Abstract] OR "neural networks"[Title/Abstract] OR "CNN"[Title/Abstract] OR "support vector machine"[Title/Abstract] OR "SVM"[Title/Abstract] OR "gradient boosting machine"[Title/Abstract] OR "GBM"[Title/Abstract] OR "Nomogram"[Title/Abstract] OR "XGBoost"[Title/Abstract] OR "Adaboost"[Title/Abstract] OR "decision tree"[Title/Abstract] OR "ResNet-50"[Title/Abstract] OR "ResNet"[Title/Abstract] OR "Radiomics"[Title/Abstract] OR "Radiomic"[Title/Abstract] OR "naive bayesian"[Title/Abstract] OR "Risk-Prediction"[Title/Abstract] OR "Risk-Prediction"[Title/Abstract] | 360,046 | 2:35:38 |
| 1 | "Machine Learning"[Mesh] | Most Recent |  | "Machine Learning"[MeSH Terms] | 64,344 | 2:28:42 |

**2.WOS 6414**

| Entitlements | # | Search Query | Database | Results | Date Run |
| --- | --- | --- | --- | --- | --- |
| - WOS.IC: 1993 to 2024 - WOS.CCR: 1985 to 2024 - WOS.SCI: 1975 to 2024 - WOS.AHCI: 1975 to 2024 - WOS.BHCI: 2005 to 2024 - WOS.BSCI: 2005 to 2024 - WOS.ESCI: 2019 to 2024 - WOS.ISTP: 1990 to 2024 - WOS.SSCI: 1965 to 2024 - WOS.ISSHP: 1990 to 2024 | 1 | machine learning (Topic) OR Transfer Learning (Topic) OR Deep learning (Topic) OR Ensemble Learning (Topic) OR artificial intelligence (Topic) OR Prediction model (Topic) OR Prediction models (Topic) OR random forest (Topic) OR neural network (Topic) OR neural networks (Topic) OR CNN (Topic) OR Support vector machine (Topic) OR SVM (Topic) OR Gradient Boosting Machine (Topic) OR GBM (Topic) OR Nomogram (Topic) OR XGBoost (Topic) OR Adaboost (Topic) OR Decision tree (Topic) OR ResNet-50 (Topic) OR ResNet (Topic) OR Radiomics (Topic) OR Radiomic (Topic) OR Naive Bayesian (Topic) OR Risk Prediction (Topic) OR Risk-Prediction (Topic) | Web of Science Core Collection | 2352853 | Thu Feb 08 2024 16:49:17 GMT+0800 (China Standard Time) |
| - WOS.IC: 1993 to 2024 - WOS.CCR: 1985 to 2024 - WOS.SCI: 1975 to 2024 - WOS.AHCI: 1975 to 2024 - WOS.BHCI: 2005 to 2024 - WOS.BSCI: 2005 to 2024 - WOS.ESCI: 2019 to 2024 - WOS.ISTP: 1990 to 2024 - WOS.SSCI: 1965 to 2024 - WOS.ISSHP: 1990 to 2024 | 2 | Cognitive Dysfunction (Topic) OR Cognitive Dysfunctions (Topic) OR Cognitive Impairments (Topic) OR Cognitive Impairment (Topic) OR Cognitive Disorder (Topic) OR Cognitive Disorders (Topic) OR Cognitive Decline (Topic) OR Cognitive Declines (Topic) OR Mental Deterioration (Topic) OR Mental Deteriorations (Topic) OR cognitive defect (Topic) OR cognitive defects (Topic) OR cognitive deficit (Topic) OR cognitive disability (Topic) | Web of Science Core Collection | 392487 | Thu Feb 08 2024 16:51:58 GMT+0800 (China Standard Time) |
| - WOS.IC: 1993 to 2024 - WOS.CCR: 1985 to 2024 - WOS.SCI: 1975 to 2024 - WOS.AHCI: 1975 to 2024 - WOS.BHCI: 2005 to 2024 - WOS.BSCI: 2005 to 2024 - WOS.ESCI: 2019 to 2024 - WOS.ISTP: 1990 to 2024 - WOS.SSCI: 1965 to 2024 - WOS.ISSHP: 1990 to 2024 | 3 | Alzheimer Disease (Topic) OR Alzheimer Dementia (Topic) OR Alzheimer Dementias (Topic) OR Alzheimer's Disease (Topic) OR Senile Dementia (Topic) OR Alzheimer Type Dementia (Topic) OR Primary Senile Degenerative Dementia (Topic) OR Alzheimer Sclerosis (Topic) OR Alzheimer Syndrome (Topic) OR Alzheimer's Diseases (Topic) OR Alzheimer Diseases (Topic) OR Alzheimers Diseases (Topic) OR Presenile Dementia (Topic) | Web of Science Core Collection | 302466 | Thu Feb 08 2024 16:54:17 GMT+0800 (China Standard Time) |
| - WOS.IC: 1993 to 2024 - WOS.CCR: 1985 to 2024 - WOS.SCI: 1975 to 2024 - WOS.AHCI: 1975 to 2024 - WOS.BHCI: 2005 to 2024 - WOS.BSCI: 2005 to 2024 - WOS.ESCI: 2019 to 2024 - WOS.ISTP: 1990 to 2024 - WOS.SSCI: 1965 to 2024 - WOS.ISSHP: 1990 to 2024 | 4 | #1 AND #2 AND #3 | Web of Science Core Collection | 6414 | Thu Feb 08 2024 16:54:32 GMT+0800 (China Standard Time) |

**3.Embase 10539**

| No. | Query | Results | Date |
| --- | --- | --- | --- |
| #10 | #3 AND #6 AND #9 | 10539 | 8-Feb-24 |
| #9 | #7 OR #8 | 262096 | 8-Feb-24 |
| #8 | 'alzheimer disease':ab,ti OR 'alzheimer dementia':ab,ti OR 'alzheimer dementias':ab,ti OR 'alzheimers disease':ab,ti OR 'senile dementia':ab,ti OR 'alzheimer type dementia':ab,ti OR 'primary senile degenerative dementia':ab,ti OR 'alzheimer sclerosis':ab,ti OR 'alzheimer syndrome':ab,ti OR 'alzheimer diseases':ab,ti OR 'alzheimers diseases':ab,ti OR 'presenile dementia':ab,ti | 33255 | 8-Feb-24 |
| #7 | 'alzheimer disease'/exp | 255471 | 8-Feb-24 |
| #6 | #4 OR #5 | 673260 | 8-Feb-24 |
| #5 | 'cognitive defect':ab,ti OR 'cognitive dysfunction':ab,ti OR 'cognitive dysfunctions':ab,ti OR 'cognitive impairments':ab,ti OR 'cognitive impairment':ab,ti OR 'cognitive disorder':ab,ti OR 'cognitive disorders':ab,ti OR 'cognitive decline':ab,ti OR 'cognitive declines':ab,ti OR 'mental deterioration':ab,ti OR 'mental deteriorations':ab,ti OR 'cognitive defects':ab,ti OR 'cognitive deficit':ab,ti OR 'cognitive disability':ab,ti | 209158 | 8-Feb-24 |
| #4 | 'cognitive defect'/exp | 635627 | 8-Feb-24 |
| #3 | #1 OR #2 | 721507 | 8-Feb-24 |
| #2 | 'machine learning':ab,ti OR 'transfer learning':ab,ti OR 'deep learning':ab,ti OR 'ensemble learning':ab,ti OR 'artificial intelligence':ab,ti OR 'prediction model':ab,ti OR 'prediction models':ab,ti OR 'random forest':ab,ti OR 'neural network':ab,ti OR 'neural networks':ab,ti OR cnn:ab,ti OR 'support vector machine':ab,ti OR svm:ab,ti OR 'gradient boosting machine':ab,ti OR gbm:ab,ti OR nomogram:ab,ti OR xgboost:ab,ti OR adaboost:ab,ti OR 'decision tree':ab,ti OR 'resnet 50':ab,ti OR resnet:ab,ti OR radiomics:ab,ti OR radiomic:ab,ti OR 'naive bayesian':ab,ti OR 'risk prediction':ab,ti OR 'risk prediction':ab,ti | 496045 | 8-Feb-24 |
| #1 | 'machine learning'/exp | 449957 | 8-Feb-24 |

**4.Cochrane 248**

Search Name: AD 2024

Date Run: 08/02/2024 09:20:33

Comment:

ID Search Hits

#1 MeSH descriptor: [Machine Learning] explode all trees 951

#2 (machine learning):ti,ab,kw OR (Transfer Learning):ti,ab,kw OR (Deep learning):ti,ab,kw OR (Ensemble Learning):ti,ab,kw OR (artificial intelligence):ti,ab,kw 6942

#3 (Prediction model):ti,ab,kw OR (Prediction models):ti,ab,kw OR (random forest):ti,ab,kw OR (neural network):ti,ab,kw OR (neural networks):ti,ab,kw 10988

#4 (CNN):ti,ab,kw OR (Support vector machine):ti,ab,kw OR (SVM):ti,ab,kw OR (Gradient Boosting Machine):ti,ab,kw OR (GBM):ti,ab,kw 2057

#5 (Nomogram):ti,ab,kw OR (XGBoost):ti,ab,kw OR (Adaboost):ti,ab,kw OR (Decision tree):ti,ab,kw OR (ResNet-50):ti,ab,kw 2712

#6 (ResNet):ti,ab,kw OR (Radiomics):ti,ab,kw OR (Radiomic):ti,ab,kw OR (Naive Bayesian):ti,ab,kw OR (Risk Prediction):ti,ab,kw 7372

#7 (Risk-Prediction):ti,ab,kw 1067

#8 #1 OR #2 OR #3 OR #4 OR #5 OR #6 OR #7 21962

#9 MeSH descriptor: [Cognitive Dysfunction] explode all trees 3818

#10 (Cognitive Dysfunction):ti,ab,kw OR (Cognitive Dysfunctions):ti,ab,kw OR (Cognitive Impairments):ti,ab,kw OR (Cognitive Impairment):ti,ab,kw OR (Cognitive Disorder):ti,ab,kw 42024

#11 (Cognitive Disorders):ti,ab,kw OR (Cognitive Decline):ti,ab,kw OR (Cognitive Declines):ti,ab,kw OR (Mental Deterioration):ti,ab,kw OR (Mental Deteriorations):ti,ab,kw 30429

#12 (cognitive defect):ti,ab,kw OR (cognitive defects):ti,ab,kw OR (cognitive deficit):ti,ab,kw OR (cognitive disability):ti,ab,kw 13578

#13 #9 OR #10 OR #11 OR #12 57997

#14 MeSH descriptor: [Cognitive Dysfunction] explode all trees 3818

#15 (Alzheimer Disease):ti,ab,kw OR (Alzheimer Dementia):ti,ab,kw OR (Alzheimer Dementias):ti,ab,kw OR (Alzheimer's Disease):ti,ab,kw OR (Senile Dementia):ti,ab,kw 14010

#16 (Alzheimer Type Dementia):ti,ab,kw OR (Primary Senile Degenerative Dementia):ti,ab,kw OR (Alzheimer Sclerosis):ti,ab,kw OR (Alzheimer Syndrome):ti,ab,kw OR (Alzheimer's Diseases):ti,ab,kw 2490

#17 (Alzheimer Diseases):ti,ab,kw OR (Alzheimers Diseases):ti,ab,kw OR (Presenile Dementia):ti,ab,kw 1159

#18 #14 OR #15 OR #16 OR #17 16981

#19 #8 AND #13 AND #18 261

Table S2 Quality Assessment of Diagnostic Accuracy Studies

| No | First author | Year of publication | 1.Patient selection: could the selection of patients have introduced bias? | | | 2.Index test: could the conduct or interpretation of the index test have introduced bias? | | 3.Reference standard: Could the reference standard, its conduct, or its interpretation have introduced bias? | | 4.Flow and timing: Could the patient flow have introduced bias? | | | Applicability | | |
| --- | --- | --- | --- | --- | --- | --- | --- | --- | --- | --- | --- | --- | --- | --- | --- |
|  |  |  | Que1 | Que2 | Que3 | Que1 | Que2 | Que1 | Que2 | Que1 | Que2 | Que3 | Que1 | Que2 | Que3 |
| 1 | Yosra Kazemi | 2018 | Yes | No | Yes | No | No | Yes | Unclear | Yes | Yes | Yes | Match | High risk of bias | Applicable |
| 2 | Yu Wang | 2019 | Yes | No | Yes | No | No | Yes | Unclear | Yes | Yes | Yes | Match | Low risk of bias | Applicable |
| 3 | Bocheng Wang | 2022 | Yes | No | Yes | No | No | Yes | Unclear | Yes | Yes | Yes | Match | High risk of bias | Applicable |
| 4 | SAMAN SARRAF | 2016 | Yes | No | Yes | No | No | Yes | Unclear | Yes | Yes | Yes | Match | Low risk of bias | Applicable |
| 5 | Modupe Odusami | 2021 | Yes | No | Yes | No | No | Yes | Unclear | Yes | Yes | Yes | Match | High risk of bias | Applicable |
| 6 | ZHE WANG | 2018 | Yes | No | Yes | No | No | Yes | Unclear | Yes | Yes | Yes | Match | Low risk of bias | Applicable |
| 7 | Harshit Parmar | 2020 | Yes | No | Yes | No | No | Yes | Unclear | Yes | Yes | Yes | Match | High risk of bias | Applicable |
| 8 | Konrad F. Waschkies | 2022 | Yes | No | Yes | No | No | Yes | Unclear | Yes | Yes | Yes | Match | Low risk of bias | Applicable |
| 9 | PR. Buvaneswari | 2021 | Yes | No | Yes | No | No | Yes | Unclear | Yes | Yes | Yes | Match | Low risk of bias | Applicable |
| 10 | Nazanin Beheshti | 2022 | Yes | No | Yes | No | No | Yes | Unclear | Yes | Yes | Yes | Match | Low risk of bias | Applicable |
| 11 | Sukrit Gupta | 2019 | Yes | No | Yes | No | No | Yes | Unclear | Yes | Yes | Yes | Match | Low risk of bias | Applicable |
| 12 | Bohyun Wang | 2022 | Yes | No | Yes | No | No | Yes | Unclear | Yes | Yes | Yes | Match | Low risk of bias | Applicable |
| 13 | Doaa Mousa | 2022 | Yes | No | Yes | No | No | Yes | Unclear | Yes | Yes | Yes | Match | High risk of bias | Applicable |
| 14 | Tingting Zhang | 2021 | Yes | No | Yes | No | No | Yes | Unclear | Yes | Yes | Yes | Match | High risk of bias | Applicable |
| 15 | Mohammadmahdi Rahimiasl | 2021 | Yes | No | Yes | No | No | Yes | Unclear | Yes | Yes | Yes | Match | High risk of bias | Applicable |
| 16 | Farheen Ramzan | 2020 | Yes | No | Yes | No | No | Yes | Unclear | Yes | Yes | Yes | Match | High risk of bias | Applicable |
| 17 | Yubraj Gupta | 2020 | Yes | No | Yes | No | No | Yes | Unclear | Yes | Yes | Yes | Match | High risk of bias | Applicable |
| 18 | Seong-Jin Son | 2017 | Yes | No | Yes | No | No | Yes | Unclear | Yes | Yes | Yes | Match | Low risk of bias | Applicable |
| 19 | Ali Khazaee | 2016 | Yes | No | Yes | No | No | Yes | Unclear | Yes | Yes | Yes | Match | Low risk of bias | Applicable |
| 20 | Ali Khazaee | 2015 | Yes | No | Yes | No | No | Yes | Unclear | Yes | Yes | Yes | Match | Low risk of bias | Applicable |
| 21 | Zhuqing Long | 2023 | Yes | No | Yes | No | No | Yes | Unclear | Yes | Yes | Yes | Match | Low risk of bias | Applicable |
| 22 | Saman Sarraf | 2023 | Yes | No | Yes | No | No | Yes | Unclear | Yes | Yes | Yes | Match | Low risk of bias | Applicable |
| 23 | Ju-Hyeon Noh | 2023 | Yes | No | Yes | No | No | Yes | Unclear | Yes | Yes | Yes | Match | High risk of bias | Applicable |

Table S2 Continue

| Risk of bias | Patient selection: could the selection of patients have introduced bias? | Que1 | Was a consecutive or random sample of patients enrolled? |
| --- | --- | --- | --- |
|  |  | Que2 | Was a case–control design avoided? |
|  |  | Que3 | Did the study avoid inappropriate exclusions? |
|  | Index test: could the conduct or interpretation of the index test have introduced bias? | Que1 | Were the index tests interpreted without knowledge of the results of the reference standard? |
|  |  | Que2 | If a threshold was used, was it prespecified? |
|  | Reference standard: Could the reference standard, its conduct, or its interpretation have introduced bias? | Que1 | Is the reference standard likely to correctly classify the target condition? |
|  |  | Que2 | Were the reference standard results interpreted without knowledge of the results of the index test? |
|  | Flow and timing: Could the patient flow have introduced bias? | Que1 | Was there an appropriate interval between the index test and the reference standard? |
|  |  | Que2 | Did all patients receive a reference standard? |
|  |  | Que4 | Were all patients included in the analysis? |
| Applicability | que1 | | Do included patients and backgrounds match the review questions? |
|  | que2 | | Do reference standard, its conduct, or its interpretation match the review questions? |
|  | que3 | | Applicability of reference standard |
